# Striatal mGlu_5_-mediated synaptic plasticity is independently regulated by location-specific receptor pools and divergent signaling pathways

**DOI:** 10.1016/j.jbc.2023.104949

**Published:** 2023-06-22

**Authors:** Yuh-Jiin I. Jong, Yukitoshi Izumi, Steven K. Harmon, Charles F. Zorumski, Karen L. ÓMalley

**Affiliations:** 1 Department of Neuroscience, Washington University School of Medicine, St Louis, Missouri, USA; 2 Department of Psychiatry, Washington University School of Medicine, St Louis, Missouri, USA; 3 The Taylor Family Institute for Innovative Psychiatric Research, Washington University School of Medicine, St Louis, Missouri, USA

**Keywords:** calcium, extracellular signal-regulated kinase (ERK), G protein-coupled receptor (GPCR), metabotropic glutamate receptor 5 (mGlu_5_), mTOR complex (mTORC), neuron, signal transduction

## Abstract

Metabotropic glutamate receptor 5 (mGlu_5_) is widely expressed throughout the central nervous system and is involved in neuronal function, synaptic transmission, and a number of neuropsychiatric disorders such as depression, anxiety, and autism. Recent work from this lab showed that mGlu_5_ is one of a growing number of G protein-coupled receptors that can signal from intracellular membranes where it drives unique signaling pathways, including upregulation of extracellular signal-regulated kinase (ERK1/2), ETS transcription factor Elk-1, and activity-regulated cytoskeleton-associated protein (Arc). To determine the roles of cell surface mGlu_5_ as well as the intracellular receptor in a well-known mGlu_5_ synaptic plasticity model such as long-term depression, we used pharmacological isolation and genetic and physiological approaches to analyze spatially restricted pools of mGlu_5_ in striatal cultures and slice preparations. Here we show that both intracellular and cell surface receptors activate the phosphatidylinositol-3-kinase–protein kinase B–mammalian target of rapamycin (PI3K/AKT/mTOR) pathway, whereas only intracellular mGlu_5_ activates protein phosphatase 2 and leads to fragile X mental retardation protein degradation and *de novo* protein synthesis followed by a protein synthesis–dependent increase in Arc and post-synaptic density protein 95. However, both cell surface and intracellular mGlu_5_ activation lead to α-amino-3-hydroxy-5-methyl-4-isoxazolepropionic acid receptor GluA2 internalization and chemically induced long-term depression albeit *via* different signaling mechanisms. These data underscore the importance of intracellular mGlu_5_ in the cascade of events associated with sustained synaptic transmission in the striatum.

The G protein coupled receptor (GPCR) metabotropic glutamate receptor 5 (mGlu_5_) has been linked to dendritic spine formation, synaptogenesis, cognition, and behavior, as well as to pathological roles in disorders such as fragile X syndrome (1), autism spectrum disorder (2, 3), anxiety, and depression (4, 5). Thus, understanding how mGlu_5_ responds to a broad range of stimuli leading to changes in protein synthesis, spine morphology, and synaptic maturation has been the focus of many preclinical and clinical studies over the last decade. These studies were prompted in part by the discovery that mGlu_5_ signaling through mitogen-activated protein kinase kinase (MEK)/extracellular signal-regulated kinase (ERK) or phosphatidylinositol-3-kinase–protein kinase B–mammalian target of rapamycin (PI3K/AKT/mTOR) pathways results in dephosphorylation and inactivation of fragile X mental retardation protein (FMRP) (1). Dephosphorylation of FMRP, an RNA translational repressor, leads to a rapid and transient burst of synaptic protein synthesis before FMRP is rephosphorylated and again represses synaptic protein translation (6, 7, 8). FMRP itself undergoes *de novo* translation along with Arc whose induction can lead to α-amino-3-hydroxy-5-methyl-4-isoxazolepropionic acid (AMPA) receptor removal from the postsynaptic density resulting in long-term depression (LTD, (9, 10, 11)). Since these groundbreaking studies, this simple model has become more complex with many new autism spectrum disorder genes discovered as well as novel FMRP targets and proposed roles (12, 13, 14). Besides novel FMRP functions, we and others have found that many GPCRs including mGlu_5_ play important new roles inside the cell by signaling from intracellular membranes. For example, we have found that the majority of mGlu_5_ is found on endoplasmic reticulum (ER) and nuclear membranes (herein referred to as intracellular membranes) in the striatum, hippocampus, and cortex where, like its surface counterpart, it couples to G_q/11_/phospholipase C/inositol trisphosphate to release intracellular Ca^2+^ from the ER or nuclear lumen (15). Intracellular mGlu_5_ receptors are also activated by glutamate, which is transported into the cell *via* the EAAT3 excitatory amino acid transporter and/or the cysteine glutamate exchanger, present at the cell surface and on intracellular membranes of many cell types (16). Although earlier studies suggested that mM concentrations of glutamate were present in the cytoplasm, later studies have shown that glutamate is highly compartmentalized in mitochondria where it can enter the tricarboxylic acid cycle following conversion to alpha-ketoglutarate. Similarly, we have shown that in striatal neurons, glutamate is also sequestered in mitochondria and that the EC_50_ for glutamate activation of intracellular mGlu_5_ is ∼60 μM, a value inconsistent with mM concentrations of free glutamate within the cytoplasm. Moreover, uncaging glutamate within the neuronal soma led to a rapid mGlu_5_-mediated Ca^2+^ response further demonstrating intracellular glutamate activation of intracellular receptors (17).

mGlu_5_-LTD has been extensively studied in the CA1 area of the hippocampus where it can be induced by (S)-3,5-dihydroxyphenylglycine (DHPG) or by paired-pulse low frequency stimulation (18) leading to the internalization of AMPA receptors and a weakening of the synapse (19, 20, 21, 22, 23). These findings have resulted in DHPG being the “go-to” agent when inducing mGlu_5_-LTD (24, 25). However, DHPG activates only cell surface mGlu_5_ (16, 26, 27) as we have shown by comparing the structure, membrane-permeability, transportability, binding curves, functional uptake, and Ca^2+^ release properties of this compound (15). Thus, results derived from DHPG treatment are in essence measuring only a fraction of the mGlu_5_ receptor pool. In contrast, synaptic release of glutamate or quisqualate (Quis) treatment, both of which are transported into the cell, constitutes a more realistic, full-fledged mGlu_5_ response.

In order to characterize intracellular mGlu_5_ function, here we focused on *in vitro* and *ex vivo* striatal preparations because mGlu_5_ is expressed in all medium spiny neurons that constitute 90 to 95% of the striatum making this a uniquely homogeneous preparation. To determine whether mGlu_5_-mediated striatal synaptic plasticity pathways are the same as those used in the hippocampus and to determine whether cell surface or intracellular mGlu_5_ receptors mediate these effects, we used pharmacological isolation as well as molecular, biochemical, and physiological techniques to show that both receptor pools mediate aspects of synaptic signaling and physiology albeit by different pathways. Unexpectedly, we found that intracellular mGlu_5_ primarily uses protein synthesis–dependent, MEK/ERK pathways, whereas cell surface mGlu_5_ uses mammalian target of rapamycin complex 2 (mTORC2) to generate striatal LTD

## Results

### Activation of both intracellular and cell surface mGlu_5_ activates components of the AKT/mTOR protein synthesis pathway

To determine intracellular mGlu_5_ responses, we used pharmacological isolation to compare the effects of membrane-impermeable, nontransported drugs (*e.g.* the antagonist LY393053 and the agonist DHPG) to those of membrane-permeable drugs like the negative allosteric modulator, 2-methyl-6-(phenylethynyl)pyridine (MPEP), as well as the agonists, glutamate and Quis, which are transported across cell membranes (16). It should be noted that at higher concentrations, Quis is also an agonist at mGlu_1_, AMPA, kainate, and *N*-methyl-D-aspartate receptors. To ensure specificity of responses, previously we have shown that Quis application in the presence of ionotropic and mGlu_1_ blockers (SYM2206, 7-(hydroxyimino)-cyclopropan[b]chromen-1a-carboxylate ethyl ester [CPCCOEt], 6-cyano-7-nitroquinoxaline-2,3-dione [CNQX], or APV) still led to a rise in intracellular Ca^2+^ in WT but not mGlu_5_ KO cultures (26). These data rule out the possibility that off target receptors are mediating Quis effects.

**Figure 1:**
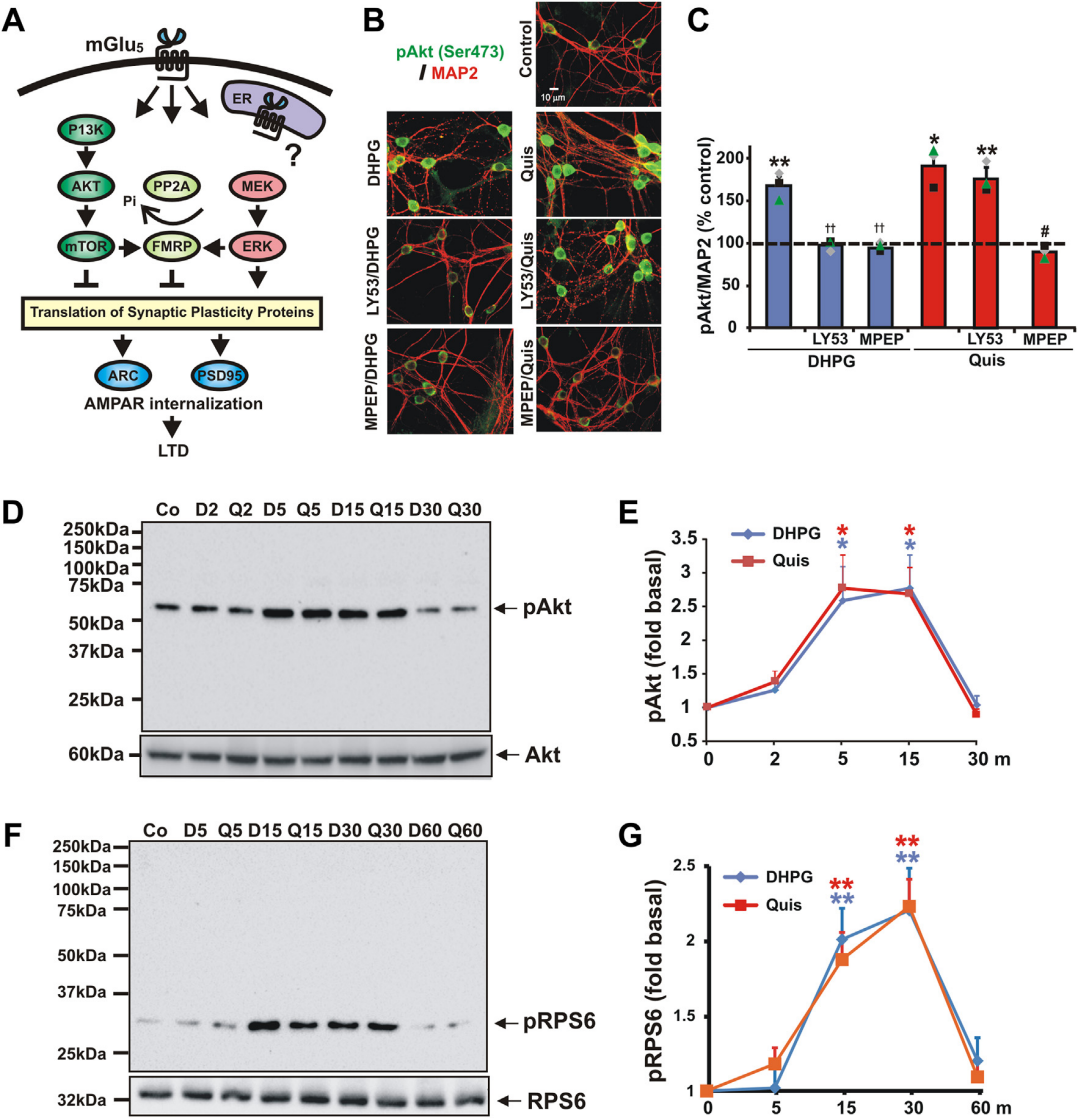
**Both DHPG and Quis increase the phosphorylation of Akt and RPS6 in striatal neurons**. *A*, model depicting DHPG-mediated mGlu_5_ pathways documented in hippocampal preparations (7, 8, 9, 10, 11, 28, 29, 42); intracellular mGlu_5_ signaling is depicted by the purple shape–labeled ER (26). *B*–*G*, striatal neurons were treated with DHPG or Quis as described in the Experimental procedures. *B* and *C*, cells were fixed after 5 min and stained with pAkt and the neuronal marker MAP2. *B*, both DHPG and Quis increased phosphorylation of pAkt (*green*) in MAP2-expressing neurons (*red*). *C*, quantification of average intensity of pAkt immunoreactivity in MAP2-positive cells. The DHPG response was blocked by LY53 and MPEP. Quis response was blocked by MPEP but not by LY53. Bars represent the mean of three experiments ± S.E.M. Individual experiments are denoted by a ▲, ■, or ♦. ∗, ∗∗ denotes statistical significance compared to control with a Student's *t* test: ∗*p* < 0.05, ∗∗*p* < 0.01 (*p* = 0.009 for DHPG-treated, *p* = 0.011 for Quis-treated, *p* = 0.009 for LY53/Quis-treated *versus* control). #denotes statistical significance compared to agonist increased levels: #*p* < 0.05 (*p* = 0.016 for LY53/DHPG- *versus* DHPG-treated, *p* = 0.015 for MPEP/Quis- *versus* Quis-treated). *D*–*G*, Western blot analysis of striatal lysates prepared from whole cells treated with DHPG or Quis. *D*, representative western blots for pAkt and total Akt after DHPG or Quis treatment. Both DHPG and Quis increased Akt phosphorylation between 5 and 15 min. *E*, quantification of Western blot data for pAkt after DHPG or Quis treatment. pAkt immunoreactivity was normalized to total Akt immunoreactivity. Line graph shows mean ± S.E., n = 3. ∗denotes statistical significance compared to control: ∗*p* < 0.05 (*p* = 0.025 for DHPG at 5 min, *p* = 0.020 for Quis at 5 min, *p* = 0.035 for DHPG at 15 min, *p* = 0.034 for Quis at 15 min treatment *versus* control). *F*, representative western blots for pRPS6 and total RPS6 after DHPG or Quis treatment. Phosphorylation of RPS6 was increased after 15 min and peaked at 30 min after DHPG or Quis treatment. *G*, quantification of Western blot data for pRPS6 after DHPG or Quis treatment. Line graph shows mean ± S.E., n = 3. ∗∗denotes statistical significance compared to control: ∗∗*p* < 0.01 (*p* = 0.002 for DHPG at 15 min, *p* = 0.003 for DHPG at 30 min, *p* = 0.004 for Quis at 15 min, *p* = 0.007 for Quis at 30 min treatment *versus* control). ER, endoplasmic reticulum; mGlu5, metabotropic glutamate receptor 5; Quis, quisqualate; RPS6, ribosomal protein S6.

Using these tools, previously we showed that in striatal neurons, cell surface–localized and intracellular mGlu_5_ are associated with distinct patterns of Ca^2+^ release such that cell surface receptors exhibited rapid transient Ca^2+^ responses, whereas intracellular mGlu_5_ exhibited sustained Ca^2+^ signals (16, 26). Because hippocampal studies have highlighted several interconnecting pathways associated with mGlu_5_-dependent LTD including protein phosphatase 2 (PP2A)-dependent activation of FMRP (7, 8) and the AKT/mTOR (28) and MEK/ERK-dependent protein synthesis pathways ((29); Fig. 1*A*), we tested whether these same pathways are used in the striatum and whether they are differentially regulated by receptor-specific subcellular localization.

From *in situ* immunostaining as well as Western blotting of postnatal P1 striatal cultures following agonist treatment, Figure 1 shows that both DHPG and Quis lead to enhanced phosphorylation of AKT as well as ribosomal protein S6 (RPS6), well known markers of the AKT/mTOR pathway. Specifically, DHPG increased pAkt to 167 ± 11.8% *versus* control, and Quis increased pAkt to 190.2 ± 16.4% *versus* control (Fig. 1, *B* and *C*). Quis alone or Quis in the presence of LY393053 also led to mGlu_5_-mediated phosphorylation of these signaling components (Fig. 1). Consistent with our earlier data showing that Quis activates both cell surface and intracellular receptors, the cell surface–only antagonist LY393053 only blocked DHPG-activated responses, not those of Quis (Fig. 1). Western blotting exhibited a similar effect within striatal lysates following DHPG or Quis treatment. pAkt increased to 2.60 ± 0.50 or 2.78 ± 0.46 fold compared to control after 5 or 15 min DHPG treatment. Similarly, pAkt increased to 2.78 ± 0.51 or 2.69 ± 0.4 fold compared to control after 5 or 15 min Quis treatment. In agreement with the hippocampal results (27), phosphorylation of AKT exhibited a faster rise than RPS6, peaking 5 to 15 min after treatment and then falling to baseline by 30 min. Phosphorylation of RPS6 began rising between 5 and 15 min after treatment, peaked at 30 min, then fell to baseline at 60 min. pRPS6 increased to 2.06 ± 0.21 or 2.20 ± 0.27 fold compared to control after 15 or 30 min DHPG treatment. Similarly, pRPS6 increased to 1.88 ± 0.18 or 2.23 ± 0.18 fold compared to control after 15 or 30 min Quis treatment (Fig. 1, *D*–*G*). In either case, there were no significant differences in agonist amplitude or the time course of response. These findings indicate that either cell surface or intracellular mGlu_5_ receptor pools can activate AKT/mTOR pathways in striatal neurons.

### Activation of intracellular but not cell surface mGlu_5_ activates PP2A phosphatase, decreases FMRP levels, and enhances protein synthesis in striatal neurons

**Figure 2:**
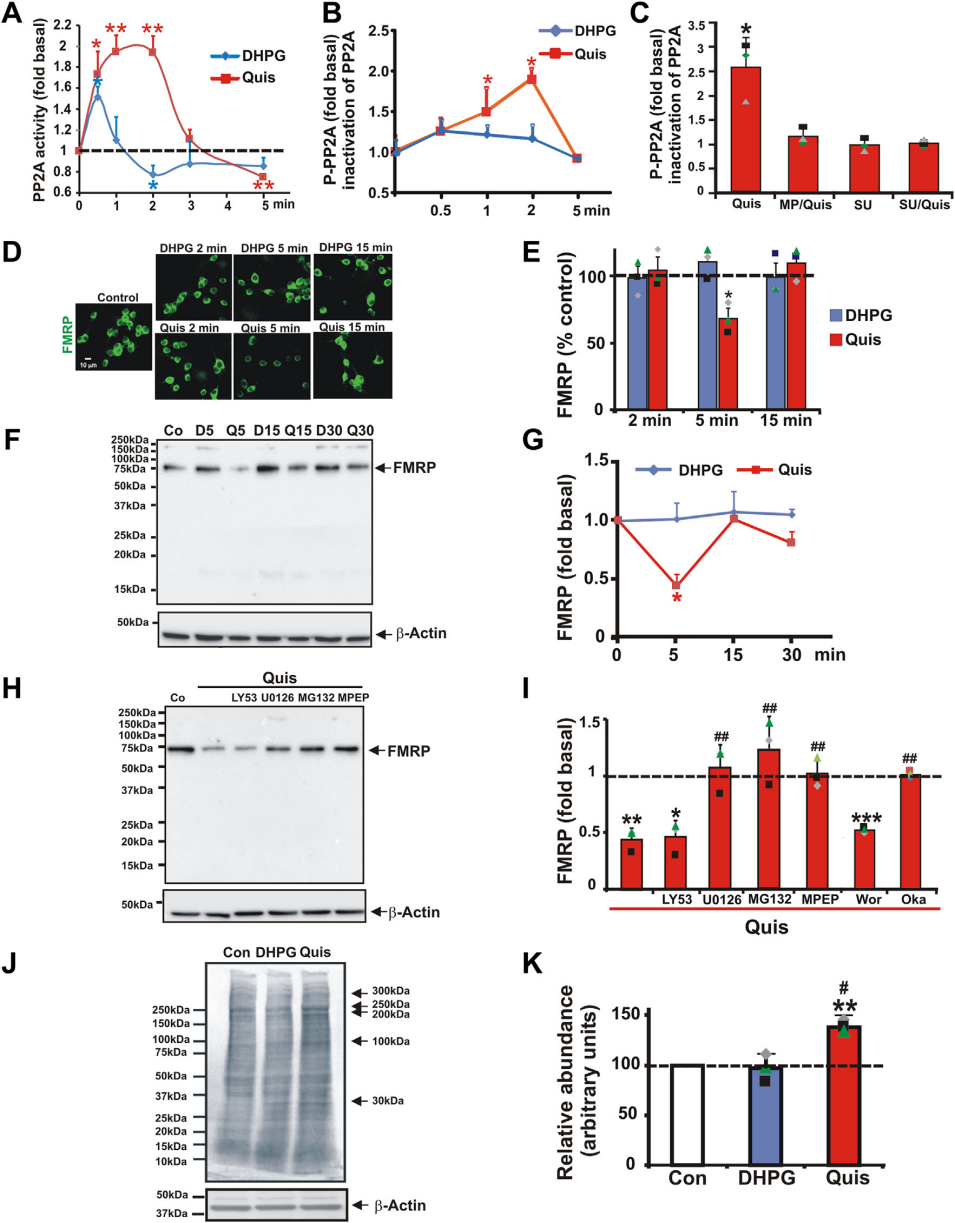
**Treatment with Quis but not DHPG leads to increased PP2A activity, decreased FMRP expression levels, and increased protein synthesis.***A*, striatal neurons were treated with DHPG or Quis for 0.5, 1, 2, 3, or 5 min. Neuronal lysates revealed that Quis generated a ∼2-fold increase of PP2A activity between 0.5 and 2 min, while DHPG generated a small, transient increase at 0.5 min. PP2A activity decreased below baseline after 2 min DHPG treatment and 5 min Quis treatment (n = 3). Student's *t* test was used to determine the significance of the fold change at different time points compared to t = 0. ∗, ∗∗denotes statistical significance compared to control: ∗*p* < 0.05, ∗∗*p* < 0.01 (*p* = 0.017 for 0.5 min DHPG, *p* = 0.026 for 2 min DHPG, *p* = 0.017 for 0.5 min Quis, *p* = 0.003 for 1 min Quis, *p* = 0.003 for 2 min Quis, *p* = 0.0097 for Quis 5 min). *B*, quantification of Western blot data for phospho-PP2A (P-PP2A-Y307) reflecting PP2A inactivation after DHPG or Quis treatment. Line graph shows mean ± S.E., n = 3. *C*, quantitative analysis of western blots after 2 min Quis treatment revealed that MPEP and the Src kinase inhibitor SU6656 blocked PP2A Y307 phosphorylation. *D*ER, endoplasmic reticulum; *I*, striatal cultures were treated with DHPG or Quis for indicated time. *D*, cells were fixed and stained with an anti-FMRP antibody. DHPG did not affect FMRP staining, while Quis induced a transient decrease in FMRP staining at 5 min. *E*, quantification of average intensity of FMRP immunoreactivity in striatal neurons. FMRP staining decreased to 68 ± 7.9% compared to control after 5 min Quis treatment. ∗denotes statistical significance compared to control: ∗*p* < 0.05 (*p* = 0.019 for 5 min Quis treatment *versus* control). *F*–*I*, Western blot analysis of striatal lysates treated with DHPG or Quis for indicated time. *F*, representative western blots for FMRP after DHPG or Quis treatment. DHPG did not affect FMRP expression levels, while Quis induced a transient decrease of FMRP expression after 5 min treatment. *G*, quantification of Western blot data for FMRP after DHPG or Quis treatment. FMRP immunoreactivity was normalized to β-actin immunoreactivity. Quis reduced FMRP levels to 0.43 ± 0.10 fold compared to control after 5 min treatment. Line graph shows mean ± S.E., n = 3. ∗denotes statistical significance compared to control: ∗*p* < 0.05 (*p* = 0.032 for 5 min Quis treatment). *H*, representative western blots for FMRP after 5 min treatment with Quis in the presence of LY53, the MEK1/2 inhibitor U0126, the proteasome inhibitor MG132 or MPEP. *I*, quantitative analysis of Western blotting results in (*H*) revealed that 5 min after Quis treatment, the FMRP level was reduced to 0.45 ± 0.10 fold compared to control. U0126, MG132, and MPEP but not LY53 blocked the Quis-mediated decrease in FMRP levels. Additional experiments and quantitative analysis of western blots after 5 min Quis treatment revealed that Quis-mediated decrease of FMRP level was not affected by wortmannin (Wor). The FMRP level was reduced to 0.51 ± 0.03 fold compared to control (*p* = 0.0005 *versus* control, *p* = 0.086 *versus* Quis). However, the Quis-mediated decrease of FMRP was completely blocked by okadaic acid (Oka). The FMRP level was the same as control (0.99 ± 0.03 fold, *p* = 0.378 *versus* control, *p* = 0.002 *versus* Quis). ∗, ∗∗, ∗∗∗denotes statistical significance compared to control: ∗*p* < 0.05, ∗∗*p* < 0.01 (*p* = 0.005 for Quis, *p* = 0.011 for LY53/Quis), ∗∗∗*p* < 0.001. ##denotes statistical significance compared to Quis: ##*p* < 0.01 (*p* = 0.004 for U0126/Quis, *p* = 0.0096 for MG132/Quis, *p* = 0.0097 for MPEP/Quis *versus* Quis). *J* and *K*, activation of intracellular mGlu_5_ significantly enhances protein synthesis. Striatal cultures were treated with radiolabel and then with DHPG or Quis for 15 min. Protein lysates were separated by SDS-PAGE and transferred onto the phosphorimager plate. *H*, autoradiography of the dried membrane revealed significantly higher protein levels in the Quis-treated lysates. *K*, quantitative analysis of the results in (*J*) revealed that DHPG had no effect on protein synthesis, while Quis increased the protein synthesis to 137.2 ± 6.34%. *∗*∗denotes statistical significance compared to control: ∗∗*p* < 0.01 (*p* = 0.005 for Quis) #denotes statistical significance compared to DHPG-treated: #*p* < 0.05 (*p* = 0.018 for Quis *versus* DHPG). In all cases, bars represent the mean of three experiments ± S.E.M. Individual experiments are denoted by a ▲, ■, or ♦. FMRP, fragile X mental retardation protein; MEK, mitogen-activated protein kinase kinase; mGlu5, metabotropic glutamate receptor 5; PP2A, protein phosphatase 2; Quis, quisqualate.

Previous studies in hippocampal cultures have shown that mGlu_5_ stimulation led to the rapid activation of PP2A phosphatase activity (∼1–2 min) leading to the dephosphorylation of FMRP followed by its subsequent degradation (∼5 min; (7, 19, 30); Fig. 1*A*). As described by others (7, 31), phosphatase activity can then be rapidly suppressed by phosphorylation of Tyr^307^ of PP2A’s catalytic subunit allowing for the rephosphorylation of FMRP, a concomitant renewal of translation suppression, and a return to FMRP’s basal state (10–15 min; (7, 11, 31)). Exactly how mGlu_5_ stimulation triggers this chain of events is unclear. However, other GPCRs have been linked to PP2A activation *via* increased PKA activity (32), increased PKC activity (33), and/or increased intracellular Ca^2+^ levels (34, 35). In order to corroborate mGlu_5_-mediated PP2A activation, here we tested whether agonist stimulation upregulated striatal PP2A activity and, if so, which receptor pool was involved. Interestingly, we show that PP2A activity is primarily regulated by intracellular mGlu_5_. Quis generated a ∼2-fold increase of PP2A activity between 0.5 and 2 min, while DHPG generated a small, transient (1.51 ± 0.09 fold) increase at 0.5 min. PP2A activity decreased to 0.78 ± 0.08 fold after 2 min DHPG treatment and to 0.75 ± 0.04 fold after 5 min Quis treatment (Fig. 2*A*). By calculating the area under the curve, it appears there is a 5.6 ± 0.9–fold difference in agonist response (Fig. 2*A*). Thus, akin to the results of Narayanan *et al*. (7), in the hippocampus, we see mGlu_5_-mediated increased PP2A enzyme activity, although it is driven by intracellular receptor activation. In support of the hypoistthesis that increased intracellular Ca^2+^ can activate PP2A (34, 35), we note that PP2A activity levels are proportional to agon-driven Ca^2+^ differences we described previously (16, 26), that is, ∼5 fold difference in DHPG and Quis responses (Fig. 2*A*, and see Discussion).

PP2A activity can be rapidly suppressed by phosphorylation of Tyr^307^ of its catalytic subunit. Phosphorylation of this tyrosine has been linked to receptor tyrosine kinases in many tissues and specifically p60c-Src in the striatum (31). Indeed, Src has also been shown to form a complex with mGlu_5_ receptors (31). To test for the suppression of PP2A activity following DHPG or Quis activation and whether Src activity played a role, we used western blots to determine levels of phospho-Tyr^307^ together with appropriate inhibitors. Figure 2*B* shows that DHPG-treated lysates showed only basal levels of PP2A phospho-Tyr^307^ across a 5 min time span, whereas Quis not only induced the rapid PP2A activation peaking between 1 to 2 min (Fig. 2*A*), but its subsequent suppression *via* an increase in phospho-Tyr^307^ levels peaking in this same time frame and declining to baseline by 5 min (Fig. 2*B*). The Quis-mediated change in phospho-Tyr^307^ could be blocked by MPEP as well as the Src inhibitor, SU6656 (Fig. 2*C*). These data corroborate earlier data in hippocampal and striatal cultures but show that the major PP2A activator is intracellular mGlu_5_ in the striatum.

Inasmuch as Narayanan *et al*. (7, 8) demonstrated that PP2A activation led to FMRP dephosphorylation using a phospho-specific FMRP antibody (7, 8), we subsequently tested whether mGlu_5_ activation altered FMRP phosphorylation using several commercially available FMRP antibodies directed against phospho-S499 without success. Given the myriad reasons why antibodies might work in certain instances and not in others (*e.g.* investigator-made vs commercial preparation, commercial batch-to-batch variations and/or different cell types), we went on to test downstream signaling steps predicted to occur after FMRP dephosphorylation. For example, following PP2A activation, mGlu_5_ activation in the hippocampus leads to the rapid degradation of FMRP itself *via* the ubiquitin proteasome system (30, 36). To determine whether this aspect of FMRP regulation was affected by a particular mGlu_5_ receptor pool in the striatum *versus* the hippocampus, we again used *in situ* immunostaining and Western blotting of striatal cultures following agonist treatment over a 15 min time course. Figure 2, *D*–*G* shows that only Quis promoted rapid degradation of FMRP within 5 min of treatment; FMRP returned to baseline levels within 15 min. Quis-mediated degradation of FMRP was blocked by MPEP but not LY393053 (Fig. 2, *H* and *I*). Quis-induced declines in FMRP were also inhibited by the proteasome inhibitor, MG132, the MEK/ERK blocker, U0126 (Fig. 2, *H* and *I*), as well as the PP2A inhibitor, okadaic acid, but not by the PI3K inhibitor (wortmannin) (Fig. I). Taken together, these data are consistent with the notion that FMRP is dynamically regulated by intracellular but not cell surface mGlu_5_ in the striatum.

Numerous studies in hippocampal preparations have shown that loss of FMRP activity *via* dephosphorylation or degradation leads to increased translation of stalled synaptic mRNAs (37, 38, 39). To examine whether protein synthesis is elevated under conditions where FMRP activity or expression levels are decreased and hence no longer able to prevent protein synthesis, we performed metabolic labeling following agonist treatment of striatal cultures. Our results reveal a significant increase of basal protein synthesis in striatal lysates following Quis but not DHPG treatment for 15 min compared with control (Fig. 2, *J* and *K*). The magnitude of the protein synthesis increase is the same if not higher than that previously reported in hippocampal preparations (*e.g.* (29)). Besides the location-specific differences, these results suggest there are cell type–specific differences as well since in the striatum, only the Quis-treated pool of receptors increased protein synthesis whereas DHPG is necessary for elevated protein synthesis in hippocampal slices.

### Intracellular but not cell surface mGlu_5_ activation increases expression of Arc in striatal neurons

**Figure 3:**
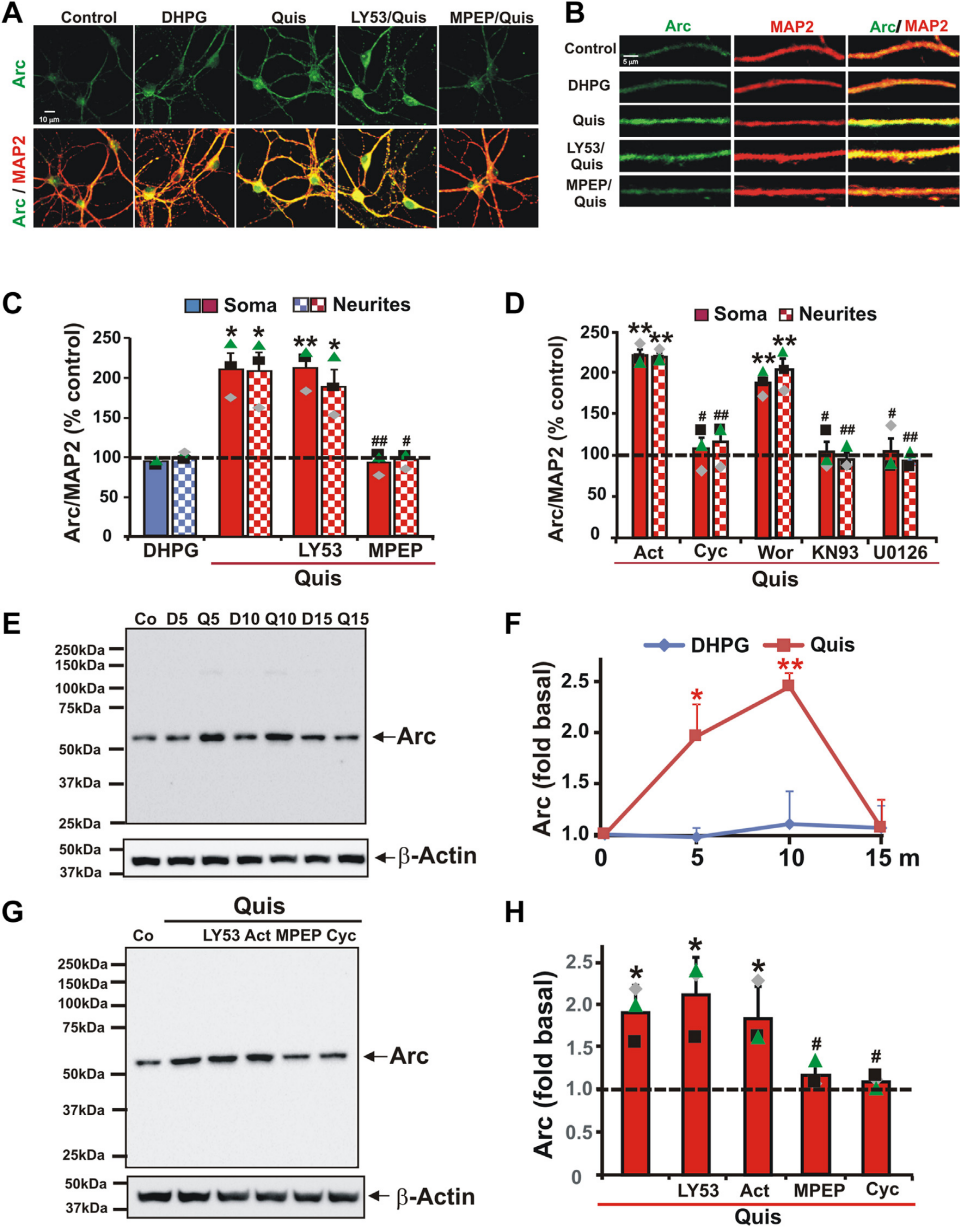
**Arc is upregulated by activation of intracellular mGlu**_**5**_. *A*, representative images of striatal neurons treated with different mGlu_5_ agonists and antagonists costained with Arc (*green*) and MAP2 (*red*). *B*, enlarged view of neurites 50 μm from cell body demonstrating increased Arc expression due to Quis compared to control. *C*, quantification of Arc staining intensity for both soma (*solid bars*) and neurites (*checked bars*) expressed as mean ± S.E.M. Individual experiments are denoted by a ▲, ■, or ♦. Quis but not DHPG increased Arc staining in cell bodies and neurites. MPEP but not LY53 blocked Quis-increased Arc levels. Multiple coverslips with more than 100 soma and neurites were analyzed per treatment in each experiment, N = 3. ∗, ∗∗denotes statistical significance compared to control with a Student's *t* test: ∗*p* < 0.05, ∗∗*p* < 0.01 (*p* = 0.015 for Quis-treated soma, *p* = 0.022 for Quis-treated neurites, *p* = 0.009 for LY53/Quis-treated soma, *p* = 0.026 for LY53/Quis-treated neurites *versus* basal level). #, ## denotes statistical significance compared to Quis-treated: #*p* < 0.05, ##*p* < 0.01 (*p* = 0.006 for MPEP/Quis-treated soma, *p* = 0.013 for MPEP/Quis-treated neurites *versus* Quis-treated level). *D*, quantification of Arc staining was as described in (*C*). Striatal cultures were preincubated with cyclohexylamine (Cyc), actinomycin D (Act), wortmannin (Wor), KN93, or U0126 for 30 min before applying Quis for 5 min. Act and Wor did not block Quis-induced Arc staining, but neurons treated with Cyc, KN93, or U1026 abolished the Quis-induced Arc staining. ∗, ∗∗denotes statistical significance compared to control: ∗*p* < 0.05, ∗∗*p* < 0.01 (*p* = 0.002 for Act/Quis soma, *p* = 0.001 for Act/Quis neurites, *p* = 0.005 for Wor/Quis soma, *p* = 0.008 for Wor/Quis neurites *versus* basal level). #, ##denotes statistical significance compared to Quis-treated neurons: #*p* < 0.05, ## *p* < 0.01 (*p* = 0.022 for Cyc/Quis soma, *p* = 0.007 for Cyc/Quis neurites, *p* = 0.028 for KN93/Quis soma, *p* = 0.007 for KN93/Quis neurites, *p* = 0.033 for U0126/Quis soma, *p* = 0.001 for U0126/Quis neurites *versus* Quis-treated soma or neurites). *E*–*H*, Western blot analysis of striatal lysates treated with DHPG or Quis for indicated times. *F*, quantification of Western blot data for FMRP after DHPG or Quis treatment. Quis increased Arc expression at 5 and 10 min, while DHPG had no effect on Arc basal level at each indicated time. Levels of Arc immunoreactivity were normalized to β-actin levels. Line graph shows mean ± S.E., n = 3. ∗denotes statistical significance compared to control: ∗*p* < 0.05, ∗∗*p* < 0.01 (*p* = 0.012 for Quis at 5 min, *p* = 0.002 for Quis at 10 min *versus* control). *G*, representative western blots for Arc after 5 min treatment with Quis in the presence of different inhibitors. *H*, quantitative analysis of Western blotting results in (*G*). Bars represent the mean of three independent experiments ± S.E. Individual experiments are denoted by a ▲, ■, or ♦. ∗denotes statistical significance compared to control: ∗*p* < 0.05 (*p* = 0.020 for Quis, *p* = 0.023 for LY53/Quis, *p* = 0.031 for Act/Quis *versus* control level), #denotes statistical significance compared to Quis: #*p* < 0.05 (*p* = 0.029 for MPEP/Quis, *p* = 0.030 for Cyc/Quis *versus* Quis increased levels). Arc, activity-regulated cytoskeleton-associated protein; FMRP, fragile X mental retardation protein; mGlu5, metabotropic glutamate receptor 5; Quis, quisqualate.

Using bioinformatics, pharmacology, and genetics, we previously showed that intracellular mGlu_5_ activated the MEK/ERK pathway in striatal cultures, a response that was blocked by MPEP but not LY393053 (26). Activation of the intracellular receptor also upregulates a number of genes in the striatum including Arc (40). Specifically, Arc mRNA was differentially activated ∼3-fold peaking at 2 h in striatal neurons; striatal Arc protein increases were most evident in neuronal nuclei and cell bodies and were blocked by pretreatment with the transcription suppressor, actinomycin D (40). Given that DHPG elevated Arc expression in hippocampal neurons within ∼15 min, we re-examined Arc induction in the striatum following agonist treatment looking at shorter time periods commensurate with synaptic protein synthesis. Extending our previous results, we found that DHPG did not increase Arc expression at 5, 10, or 15 min in striatal neurons or striatal lysates, whereas after 5 min Quis treatment, Arc soma staining increased to 210 ± 19.8% and Arc neurites staining increased to 208 ± 23.9% compared to control neurons (Fig. 3, *A*–*C*). Similarly, Arc expression was increased to 1.96 ± 0.32 or 2.48 ± 0.08 fold respectively in striatal lysates after 5 min or 10 min Quis treatment (Fig. 3, *E* and *F*). Quis-mediated increases in Arc could be blocked by pretreatment with MPEP, the protein synthesis inhibitor cycloheximide, the calcium/calmodulin-dependent protein kinase II (CaMKII) inhibitor KN93, and the MEK inhibitor, U0126, in individual neurons and neurites (Fig. 3*D*) as well as striatal lysates (Fig. 3, *G* and *H*). In contrast, inhibitors such as LY393053, actinomycin D, and wortmannin (PI3K inhibitor) were not able to block the Quis effect (Fig. 3, *D*, *G*, and *H*). These data as well as our previous results imply that Arc induction follows a biphasic curve encompassing a rapid, transient protein synthesis–dependent phase (Fig. 3) followed by a longer, more sustained response that is transcription-dependent as well (40). Both processes are CaMKII- and MEK-ERK–dependent, again revealing a cell type–specific, spatially restricted bias in mGlu_5_-mediated Arc signaling.

### Intracellular but not cell surface mGlu_5_ activation increases expression of PSD-95 in striatal neurons

**Figure 4:**
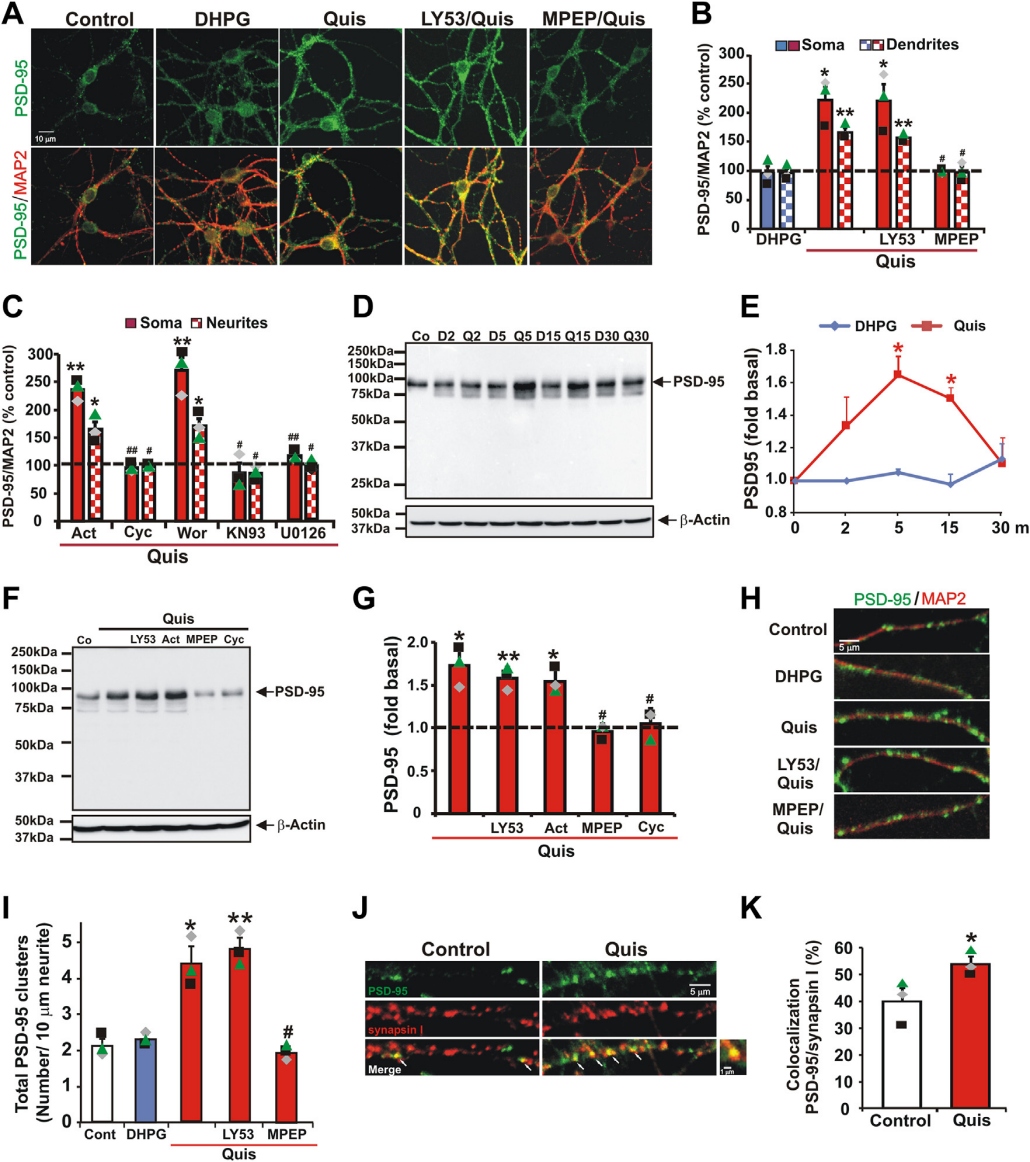
**Treatment with Quis but not DHPG leads to increased PSD-95 expression.***A–C* and *H–I*, striatal neurons were treated with DHPG or Quis and then fixed after 5 min and stained with PSD-95 (*green*) and MAP2 (*red*). *A*, representative images. *B*, quantification of immunofluorescence data. Data represent the average intensity of the PSD-95 signal as a percentage of controls in either the soma (*solid* bars) or dendrites (*checked* bars). Here and below, bars represent the mean of three experiments ± S.E.M. with more than 100 MAP2-positive neurons per treatment in each experiment analyzed; Individual experiments are denoted by a ▲, ■, or ♦. ∗denotes statistical significance compared to control determined by Student’s *t* test: ∗*p* < 0.05, ∗∗*p* < 0.01 (*p* = 0.017 for Quis-treated soma, *p* = 0.007 for Quis-treated neurites, *p* = 0.026 for LY53/Quis-treated soma, *p* = 0.002 for LY53/Quis-treated neurites *versus* control). ##denotes statistical significance compared to Quis-treated: #*p* < 0.05 (*p* = 0.020 for MPEP/Quis-treated soma, *p* = 0.015 for MPEP/Quis-treated neurites *versus* Quis-treated soma or neurites). *C*, quantification of PSD-95 immunofluorescence data as described above following preincubation with actinomycin D (Act), cyclohexylamine (Cyc), wortmannin (Wor), KN93, or U0126 for 30 min before adding Quis for 5 min. Multiple coverslips with more than 100 MAP2 positive neurons were analyzed per treatment in each experiment, N = 3. ∗, ∗∗denotes statistical significance compared to control: ∗*p* < 0.05, ∗∗*p* < 0.01 (*p* = 0.003 for Act/Quis soma, *p* = 0.025 for Act/Quis neurites, *p* = 0.009 for Wor/Quis soma, *p* = 0.019 for Wor/Quis neurites *versus* basal level). #, ##denotes statistical significance compared to Quis-treated neurons: #*p* < 0.05, ## *p* < 0.01 (*p* = 0.004 for Cyc/Quis soma, *p* = 0.010 for Cyc/Quis neurites, *p* = 0.011 for KN93/Quis soma, *p* = 0.027 for KN93/Quis neurites, *p* = 0.005 for U0126/Quis soma, *p* = 0.014 for U0126/Quis neurites *versus* Quis-treated soma or neurites). *D*–*G*, Western blot analysis of striatal lysates prepared from whole cells treated with DHPG or Quis and probed with anti-PSD-95. Levels of PSD-95 immunoreactivity were normalized to β-actin levels. Line graph shows mean ± S.E., n = 3. ∗denotes statistical significance compared to control; ∗*p* < 0.05, ∗∗*p* < 0.01 (*p* = 0.002 for Quis at 5 min, *p* = 0.017 for Quis at 10 min *versus* control). *F*, representative western blots for PSD-95 after 5 min treatment with Quis in the presence of various inhibitors (LY53, Act, MPEP, or Cyc). *G*, quantitative analysis of results in (*F*). ∗denotes statistical significance compared to control: ∗*p* < 0.05 (*p* = 0.017 for Quis, *p* = 0.008 for LY53/Quis, *p* = 0.011 for Act/Quis *versus* control level), #denotes statistical significance compared to Quis-treated: #*p* < 0.05 (*p* = 0.024 for MPEP/Quis, *p* = 0.032 for Cyc/Quis *versus* Quis). *H*, enlarged view of neurites 50 μm from cell body stained with PSD-95 (*green*) and MAP2 (*red*) after 5 min Quis treatment demonstrating increased PSD-95 cluster density due to Quis compared to control. *I*, quantitative analysis of total PSD-95 clusters per 10 μm in striatal dendrites treated with different mGlu_5_ agonists and antagonists. PSD-95 dendritic clusters were counted along 40 μm distances, and the average number of clusters per 10 μm was determined. ∗, ∗∗denotes statistical significance compared to control: ∗*p* < 0.05, ∗∗*p* < 0.01 *versus* basal level (*p* = 0.027 for Quis, *p* = 0.009 for LY53/Quis *versus* control level). #denotes statistical significance compared to Quis: #*p* < 0.05 (*p* = 0.019 for MPEP/Quis *versus* Quis increased level). *J* and *K*, DIV 14 striatal neurons were treated with DHPG or Quis for 5 min and stained with a presynaptic marker synapsin I and a postsynaptic marker PSD-95. Synaptic clusters were determined by overlaying the PSD-95 staining (*green*) with the synapsin I staining (*red*). *J*, representative images of immunofluorescence for synapsin I and PSD-95 in control or Quis-treated neurites. *K*, apposition of stained clusters in the dendrites was quantitated. For analysis of colocalization between synapsin I (*red*) and PSD-95 clusters (*green*), images from both channels were superimposed. Synapsin I and PSD-95 clusters were considered colocalized (*white arrow*) and indicative of a putative synapse if they were overlapping by at least 0.25 μm (1.24 pixel row). ∗denotes statistical significance compared to control: ∗*p* < 0.05 (*p* = 0.016 for Quis *versus* basal level). mGlu5, metabotropic glutamate receptor 5; Quis, quisqualate.

Another key protein downstream of the mGlu_5_ signaling cascade is postsynaptic density protein 95 (PSD-95). As a scaffolding protein enriched at the PSD, PSD-95 influences synaptic strength and plasticity in part by modulating AMPA receptor endocytosis and stabilizing dendritic spines (41). Like Arc, synaptic PSD-95 synthesis in the cortex and the hippocampus is regulated by mGlu_5_-dependent dephosphorylation and degradation of FMRP (42). To determine whether intracellular mGlu_5_ also plays a role in PSD-95 induction, we performed similar experiments as with Arc. Striatal cultures were treated for 5 min with DHPG or Quis in the presence or absence of LY393053 or MPEP. Quis increased PSD-95 immunofluorescence 222 ± 3.2% in soma and 167 ± 7.7% in neurites, increases that were blocked by MPEP but not by LY53 (Fig. 4, *A* and *B*). PSD-95 staining within striatal neurons was diffused within the cell soma and dendrites with increased positive puncta seen throughout the dendritic arbor. In keeping with the notion that somatic and dendritic enhancement of PSD-95 expression was dependent upon new protein synthesis, not transcription, increased PSD-95 was blocked by cycloheximide but not actinomycin D (Fig. 4*C*). As with Arc expression, elevated PSD-95 was also blocked by the KN93 and U0126 but not by the wortmannin (Fig. 4*C*). PSD-95 upregulation by intracellular mGlu_5_ activation was further confirmed by Western blot analysis. Cellular lysates revealed that Quis treatment increased PSD-95 levels to 1.67 ± 0.12 or 1.50 ± 0.07 respectively after 5 or 15 min before returning to basal levels after 30 min (Fig. 4, *D* and *E*). To determine whether the increased Quis-induced PSD-95 expression was dependent on translation or transcription, striatal cultures were either pre-incubated with cycloheximide or actinomycin D for 30 min before a 5-min Quis application. As quantitated in individual neurons and dendrites, Quis-induced PSD-95 expression in pooled lysates of 1 million cells was blocked by cycloheximide but not actinomycin D (Fig. 4, *F* and *G*). Taken together, these findings indicate that unlike in the cortex or hippocampus where DHPG can upregulate PSD-95, in the striatum only the intracellular pool of mGlu_5_ induces PSD-95 translation-dependent expression. Quis-induced rapid translation of PSD-95 is dependent upon both CaMKII and MEK/ERK pathways but not PI3K/mTOR although the exact triggers for these signals are as yet unknown.

Given that PSD-95 is most often associated with scaffolding proteins at the PSD, we also looked for changes in PSD-95 puncta along dendritic shafts. Although punctate PSD-95 staining was evident along the dendrite in untreated or DHPG-treated cultures, Quis treatment increased total PSD-95 density from 2.11 ± 0.17/10 μm neurite to 4.38 ± 0.40/10 μm neurite (Fig. 4, *H* and *I*). Pretreating cultures with MPEP and then Quis reduced the appearance of PSD-95 puncta to control levels whereas LY53 had no effect. To assess whether clusters of PSD-95 are synaptic, we costained the cultures with synapsin I (a marker of presynaptic terminals) and quantitated their apposition. Dendritic clusters stained green (PSD-95) were counted on a magnified region of the dendrite and when costained with red (synapsin 1) appeared yellow (Fig. 4*J*). The yellow clusters were considered synaptic PSD-95. PSD-95 expression is mostly nonsynaptic in striatal neurons at 14 days *in vitro* (DIV, 40.1 ± 8.1% PSD-95 puncta colocalized with synapsin I). After treatment with Quis for 5 min, the percentage of dendritic PSD-95 puncta colocalized with synapsin I increased to 54.0 ± 4.6% (Fig. 4, *J* and *K*). These results suggest that intracellular mGlu_5_ plays an important role regulating PSD-95 expression at the synapse.

### Striatal cell surface and intracellular mGlu_5_ receptors mediate GluA2 internalization *via* different signaling pathways

**Figure 5:**
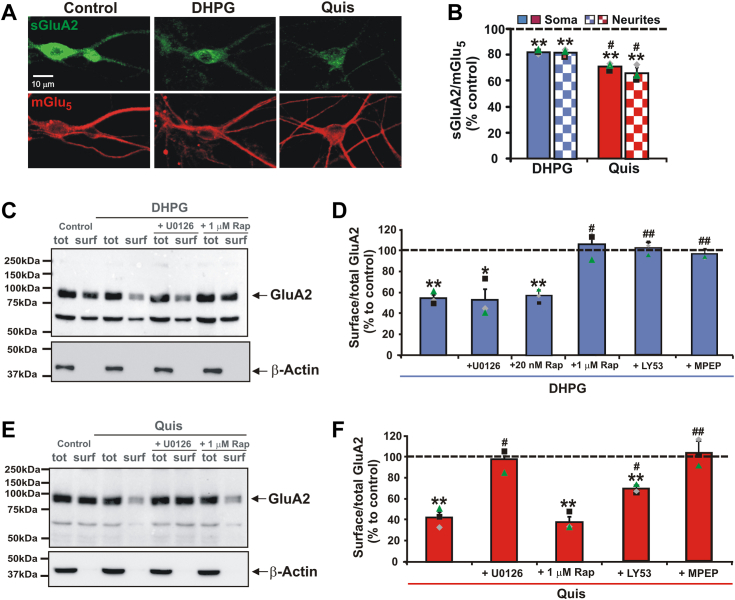
**Surface GluA2 is rapidly internalized in striatal neurons following mGlu**_**5**_**activation**. *A* and *B*, mGlu_5_ agonist treatment decreases surface GluA2 (sGluA2, *green*) staining in mGlu_5_-positive neurons (*red*). Striatal cultures were treated with DHPG or Quis for 15 min at 37 °C and stained with anti-GluA2 and anti-mGlu_5_ as described in Experimental procedures. *A*, images of sGluA2 and mGlu_5_ showing the staining from a control culture and 15 min after agonist treatment. *B*, quantitative analysis of confocal images in (*A*) revealed that 15 min following DHPG treatment, sGluA2 levels decreased to 81.7 ± 1.6% of control levels in striatal soma (*p* = 0.001 compared to control) and decreased to 81.4 ± 2.9% of control levels in striatal neurites (*p* = 0.004). Following 15 min of Quis treatment, sGluA2 levels decreased 70.5 ± 3.1% of control levels in striatal soma (*p* = 0.002 compared to control) and 65.7 ± 6.1% of control levels in striatal neurites (*p* = 0.005). Quis effects were significantly different from those of DHPG in striatal soma (*p* = 0.016) and neurites (*p* = 0.011). Here and in (*D* and *F*), bars represent the mean of three independent experiments ± S.E. Individual experiments are denoted by a ▲, ■, or ♦. ∗,∗∗denotes statistical significance (∗*p* < 0.05, ∗∗*p* < 0.01). #denotes statistical significance comparing Quis to DHPG (#*p* < 0.05, ##*p* < 0.01). *C*–*F*, Western blot analyses of total and surface GluA2 levels in striatal neurons after DHPG or Quis treatment. Cell surface proteins were labeled with cell membrane-impermeable EZ-Link Sulfo-NHS-LC-Biotin for 30 min prior to quenching and then prepared as described in the Experimental procedures. Twenty-five μg of total protein (tot) were run on a gel next to the biotinylated proteins (bio) derived from 50 μg of the equivalent neuronal lysate. β-actin served as a loading control for total lysates. *C*, representative blot showing total and biotinylated surface GluA2 from a control culture (lanes 1 and 2) and 15 min following DHPG treatment (lanes 3 and 4) in the presence of U0126 (lane 5 and 6) or 1 μΜ rapamycin (Rap, lane 7 and 8). *D*, quantitative analysis of Western blotting results in (*C*) revealed that following DHPG treatment, surface GluA2 levels were reduced to 54.0 ± 3.4% of control levels (*p* = 0.003 compared to control). U0126 did not affect DHPG-induced GluA2 internalization (52.1 ± 10.2%, *p* = 0.021 compared to control), whereas 1 μM rapamycin blocked DHPG-induced GluA2 internalization (104.9 ± 7.5%, *p* = 0.289 compared to control, *p* = 0.021 compared to DHPG). Additional experiments and quantitative analysis showed that 20 nM rapamycin did not affect DHPG-induced GluA2 internalization (56.9 ± 6.3%, *p* = 0.004 compared to control), whereas LY53 blocked this process (101.9 ± 5.9%, *p* = 0.317 compared to control, *p* = 0.009 compared to DHPG) as did MPEP (96.6 ± 5.3%, *p* = 0.194 compared to control, *p* = 0.009 compared to DHPG). *E*, representative blot showing the samples of total and biotinylated surface GluA2 from a control culture (lanes 1 and 2) and 15 min after Quis treatment (lanes 3 and 4) in the presence of U0126 (lane 5 and 6) or 1 μΜ rapamycin (lane 7 and 8). *F*, quantitative analysis of Western blotting results in (*F*) revealed that 15 min after Quis treatment, surface GluA2 levels were reduced to 41.9 ± 5.1% of control levels (*p* = 0.004 compared to control); U0126 blocked Quis-induced GluA2 internalization (97.3 ± 6.6%, *p* = 0.362 compared to control, *p* = 0.018 compared to Quis), while rapamycin did not affect Quis-induced GluA2 internalization (38.0 ± 5.0%, *p* = 0.003 compared to control). Additional experiments revealed that Quis-induced GluA2 internalization was partially blocked by LY53 (67.8 ± 4.3%, *p* = 0.003 compared to control; *p* = 0.02 compared to Quis) and completely blocked by MPEP (102.0 ± 12.6%, *p* = 0.403 compared to control, *p* = 0.007 compared to Quis). mGlu5, metabotropic glutamate receptor 5; Quis, quisqualate.

As described above, mGlu_5_ activation leads to *de novo* translation of synaptic mRNAs encoding proteins such as Arc and PSD-95, which induce LTD by decreasing surface AMPA receptors such as GluA2 in cortical and hippocampal preparations (28, 42). To determine whether this process also occurs in the striatum and which receptor pool might play a role, we tested surface staining of GluA2 in cultured striatal neurons. Both DHPG and Quis caused a decrease in surface GluA2 in mGlu_5_-positive neurons and neurites with Quis being significantly more effective than DHPG in somas (18.3% *versus* 29.5%, Fig. 5, *A* and *B*) and neurites (18.6% *versus* 34.3%, Fig. 5, *A* and *B*). To validate these findings, we conducted surface biotinylation followed by NeutrAvidin bead pull-downs and Western blot assays to measure levels of surface and total proteins. We also included pathway inhibitors to determine signaling pathways underlying GluA2 internalization. In the biotinylation assay, DHPG reduced surface GluA2 by ∼46%, an effect that could not be blocked by pretreatment with the MEK inhibitor U0126 (Fig. 5, *C* and *D*). Quis also reduced surface GluA2, in this case by ∼58%, an effect that was also blocked by U0126 (Fig. 5, *E* and *F*). All agonist effects could be blocked by the mGlu_5_ inhibitor MPEP (Fig. 5, *D* and *F*).

Although mTORC1 signaling has been reported to be necessary for mGlu_5_-LTD, a recent report suggests that mTORC2 plays the predominant role in hippocampal mGlu_5_-LTD (43). Biochemically, mTORC1 and mTORC2 are distinguished by their complex composition; besides mTOR, mTORC1 also contains raptor, which can be blocked by 20 or 200 nM rapamycin, whereas mTORC2 contains rictor (rapamycin-insensitive companion of mTOR) that makes mTORC2 largely insensitive to this agent (44). However, higher concentrations of rapamycin (1 μM) do in fact block mTORC2 (45) as well as mGlu_5_-LTD in the hippocampus (43). Interestingly, 20 nM rapamycin did not block GluA2 removal from the cell surface of DHPG-treated striatal lysates (Fig. 5*D*), whereas 1 μM rapamycin did (Fig. 5, *C* and *D*). Thus, DHPG-mediated GluA2 internalization is not dependent upon new protein synthesis (Fig. 2, *H* and *I*), Arc (Fig. 3), PSD-95 (Fig. 4), or mTORC1 but rather on mTORC2 (Fig. 5, *C* and *D*). In contrast, the ability of Quis to reduce surface GluA2 was not blocked by rapamycin (Fig. 5, *E* and *F*). All together, these results demonstrate that cell surface and intracellular mGlu_5_ receptor pools regulate GluA2 surface expression *via* distinct signaling pathways. In accordance with our previous data indicating that MEK/ERK pathway mediated many of intracellular mGlu_5_ signaling outcomes, here again MEK/ERK appears to be the signaling pathway underlying intracellular mGlu_5_ effects.

### Pharmacological isolation reveals distinct mechanisms underlying striatal mGlu_5_-LTD

**Figure 6:**
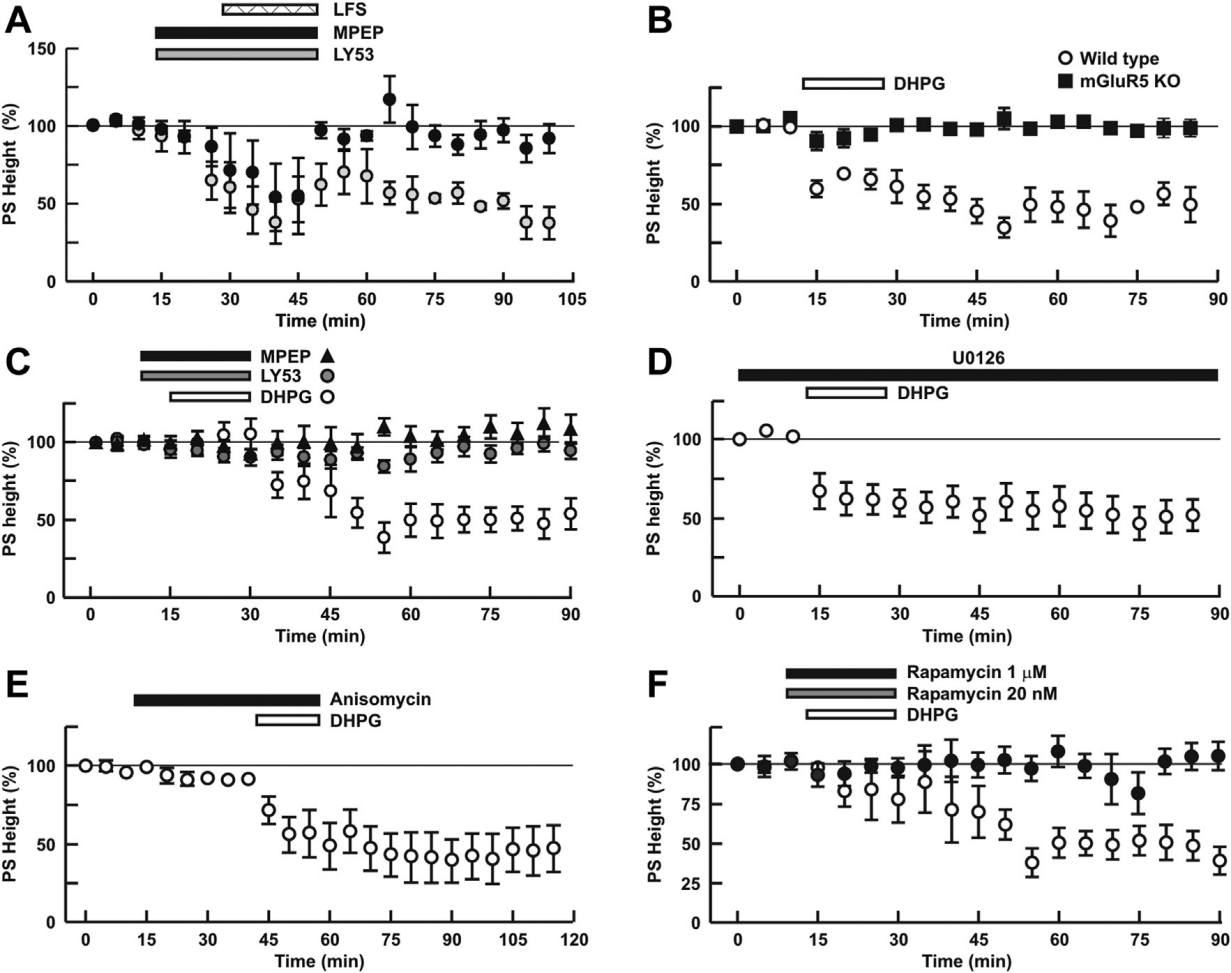
**Striatal DHPG-LTD is sensitive to rapamycin but not to anisomycin**. *A*, LFS (1 Hz × 900 pulses) induced LTD in slices from mGlu_5_ WT striata which was inhibited by MPEP (*closed bar* and *circles*) but not by LY53 (*gray bar* and *circles*). *B*, DHPG-LTD was induced by 15 min administration of DHPG (*open bar*) in slices from mGlu_5_ WT mice (*open circles*) but not from mGlu_5_ KO mice (*closed squares*). *C–F*, effects of various drugs on DHPG-LTD in slices from WT mice. In (*C*), DHPG-LTD was blocked by MPEP (*black triangles*) and LY53 (*gray circles*) in the presence of CPCCOEt, all administered 5 min prior to DHPG. *D*, U0126 was continuously administered before and during the entire recording. In (*E*), anisomycin was administered 30 min prior to DHPG, whereas the doses indicated of rapamycin (*F*) were administered 5 min prior to DHPG. *B* and *C*, average DHPG-induced LTD was significantly blocked in KO slices and by MPEP and LY53 in time-matched striatal slices (75–90 min); U0126, anisomycin, and 20 nM rapamycin did not significantly affect DHPG-LTD, whereas 1 μM completely blocked it. Values represent the mean ± SEM; n = 5 to 7 slices per treatment. mGlu5, metabotropic glutamate receptor 5; LTD, long-term depression; LFS, low frequency stimulation.

Because numerous studies have suggested that mGlu_5_ is linked with changes in hippocampal LTD, we tested whether intracellular mGlu_5_ also plays a role in this form of synaptic plasticity in the striatum. We used mGlu_5_ WT and deficient animals (46) to determine the specificity of mGlu_5_-mediated LTD in coronal striatal slices following electrical or chemical LTD paradigms. Low frequency stimulation (LFS; 1 Hz x 900 pulses) induced persistent electrical LTD, measured by monitoring population spike (PS) heights, in slices from WT mice (Fig. 6*A*). Importantly, LFS-LTD was completely inhibited by MPEP (Fig. 6*A*; closed circles; 106.8 ± 8.6%, N = 4, *p* = 0.0093 *versus* LFS alone) but not by LY-53 (gray circles; 28.2 ± 9.6%, N = 5). These results indicate that synaptically released glutamate via LFS primarily induces LTD via intracellular mGlu_5_ activation as evidenced by the response being blocked by MPEP and not by LY53. Application of DHPG itself induced LTD in P28-30 striatal slice preparations from mGlu_5_ WT mice (59.2 ± 4.9% of baseline 60 min following DHPG, N = 5), but not from mGlu_5_ KO littermates (112.0 ± 8.3% of baseline, N = 5, *p* = 0.0064 vs. DHPG alone in WT, Fig. 6*B*). Predictably, striatal DHPG-LTD in WT was blocked by both LY393053 (108.1 ± 6.6%, N = 5, *p* = 0.0129 vs. DHPG alone) and MPEP (104.1 ± 3.1%, N = 5, *p* = 0.0253 vs. DHPG alone, Fig. 6*C*). In keeping with DHPG not acting through the MEK/ERK pathway in the striatal model, U0126 had no effect on DHPG-LTD (51.2 ± 13.7%, N = 5. *p* = 0.9922 vs. DHPG alone, Fig. 6*D*). In contrast to chemical LTD induced in the hippocampus (25, 47), DHPG-LTD in striatal slices was not blocked by the protein synthesis inhibitor, anisomycin (52.7 ± 13.3%, N = 5, *p* = 0.9973 vs. DHPG alone, Fig. 6*E*), nor the mTORC1 inhibitor, low concentration rapamycin (open circles; 47.1 ± 10.5%, N = 6, *p* = 0.9179 vs. DHPG alone, Fig. 6*F*). Similar to the recent report suggesting DHPG hippocampal effects were mediated by mTORC2 (43), striatal DHPG-LTD was blocked by 1 μM rapamycin indicating mTORC2 not only affects AMPA receptor internalization but also plays a role in striatal DHPG-LTD (closed circles; 105.8 ± 11.7%, N = 7, *p* = 0.010 vs. DHPG alone, Fig. 6*F*). These data confirm and extend our findings above showing DHPG does not affect protein synthesis in striatal cultures but does induce GluA2 internalization. Although these manipulations are not specific for LTD, they support the view that new protein synthesis is not required for DHPG-LTD and that mTORC1 is not the predominant pathway underlying LTD in the striatum.

**Figure 7:**
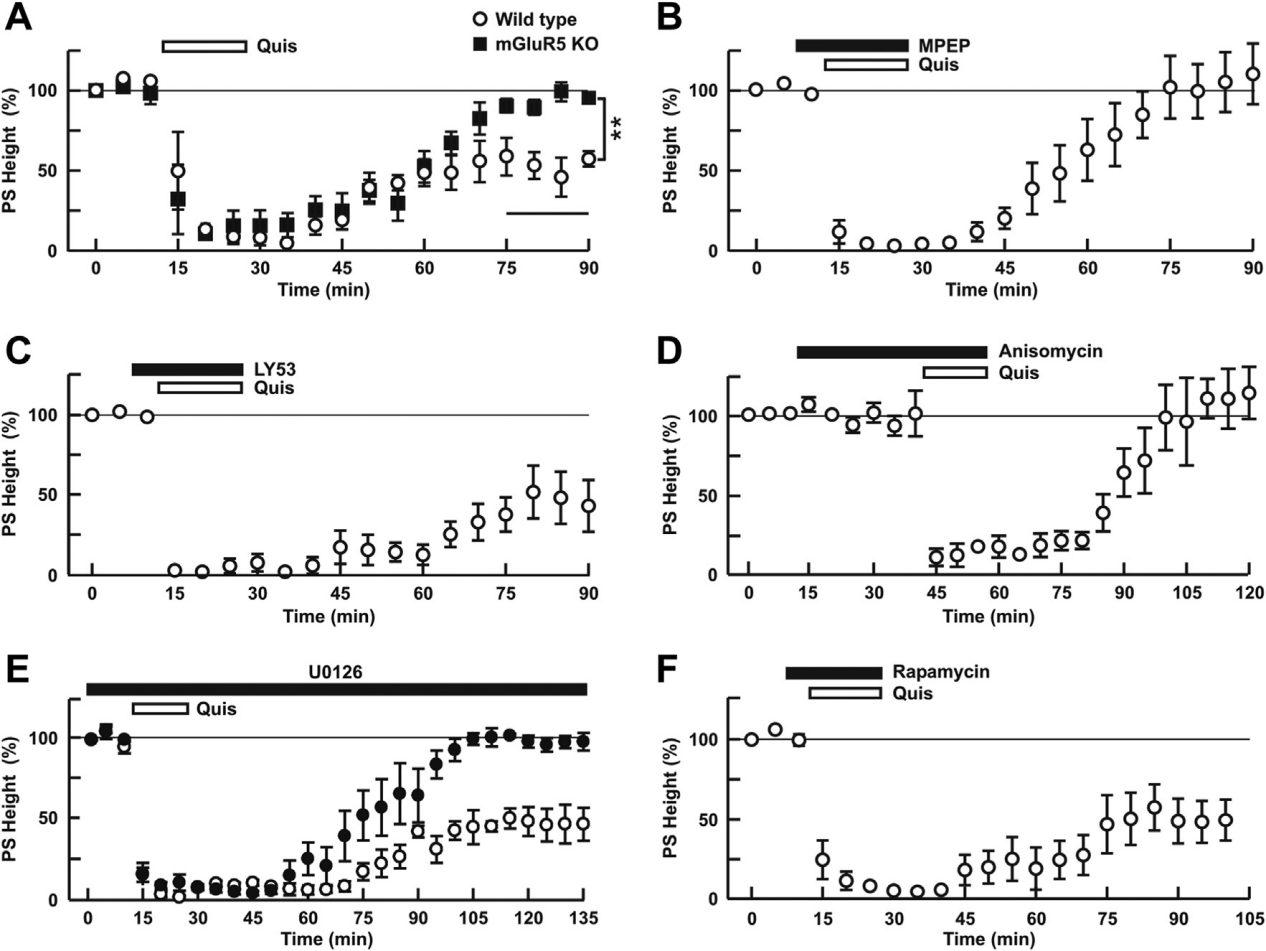
**Striatal Quis-LTD is sensitive to anisomycin but not rapamycin**. Quis-LTD was induced by 15 min administration of Quis (*open bar*). CNQX, APV, and CPCCOEt were administered 5 min prior to Quis for 20 min (*bar* is not shown). *A*, Quis induces LTD in slices from WT (*open circles*) but not from KO mice (*closed squares*). *B–F*, effects of various drugs (*black bar*) on Quis-LTD in slices from WT mice. MPEP (*B*), LY53 (*C*), and rapamycin (*F*) were administered 5 min prior to Quis, whereas anisomycin (*D*) was administered 30 min prior to Quis. U0126 (*closed circles*, *E*) was continuously administered before and during the entire recording *versus* Quis alone (*open circles*, *E*). Values represent the mean ± SEM; n = 5 to 6 slices per treatment. LTD, long-term depression; Quis, quisqualate.

We also used mGlu_5_-deficient animals to determine the specificity of Quis-LTD. Quis, administered in the presence of CNQX, APV, and CPCCOEt to block other glutamate receptors, consistently induced chemical LTD in WT (32.4 ± 11.3% of baseline, N = 5), but Quis-induced LTD was not observed in mGlu_5_ KO mice (95.6 ± 6.1%, N = 5, *p* = 0.0015 vs. Quis alone in WT, Fig. 7*A*). As predicted, application of MPEP blocked Quis-LTD in WT (111.1 ± 11.4%, N = 6, *p* < 0.0001 vs. Quis alone, Fig. 7*B*), whereas LY393053 did not (41.1 ± 8.9%, N = 5, *p* = 0.9817 vs. Quis alone, Fig. 7*C*) further emphasizing the role of intracellular mGlu_5_ in this form of synaptic plasticity. In contrast to DHPG-LTD, incubation with the translation inhibitor anisomycin blocked Quis-LTD (108.4 ± 11.4%, N = 5, *p* = 0.0001, vs. Quis alone, Fig. 7*D*) as did U0126 (109.9 ± 7.8%, N = 5. *p* = 0.000 vs. Quis alone, 53.3 ± 4.0%, Fig. 7*E*) but not high dose rapamycin (43.8 ± 14.7%, N = 5, *p* = 0.9377 vs. Quis alone, Fig. 7*F*). In the presence of U0126, the recovery after Quis administration was slow possibly due to the lipophilic nature of this compound. Thus, we monitored slices for an additional 45 min to make sure the responses had stabilized. Taken together, these results indicate that depending upon which mGlu_5_ receptor pool is activated, distinct forms of LTD are generated that are sensitive to unique signaling mechanisms.

## Discussion

While most studies of synaptic plasticity have focused on cell surface mGlu_5_ receptors, here we demonstrate a critical diversification of mGlu_5_ signaling based on its subcellular localization and the spatiotemporal output of its downstream effectors. Using pharmacological isolation, genetic and biochemical tools, we show that activation of both cell surface and intracellular pools of mGlu_5_ lead to activation of key components of the mTOR pathway, whereas activation of intracellular mGlu_5_ rapidly leads to increased PP2A activity, FMRP degradation, and enhanced protein synthesis. Moreover, intracellular mGlu_5_ activation also increases Arc and PSD95 in a translation- but not transcription-dependent manner that is blocked by CaMKII and MEK/ERK inhibition. Functional diversification of signaling based on subcellular localization of receptors is further demonstrated by our findings that both receptor pools contribute to GluA2 internalization albeit by different pathways. Activation of cell surface mGlu_5_ internalizes GluA2 in an apparent mTORC2-dependent pathway, whereas intracellular mGlu_5_ activation internalizes GluA2 in a MEK/ERK-mediated process. These unique GluA2 internalization pathways are similarly utilized in mGlu_5_ models of synaptic plasticity wherein cell surface mGlu_5_-LTD is mediated by mTORC2 but not MEK/ERK or new protein synthesis, whereas intracellular mGlu_5_-LTD requires new protein synthesis and is sensitive to MEK/ERK activation but does not rely on mTORC2. Taken together, these data suggest a major role for intracellular mGlu_5_ in the weakening of striatal synapses and underscore localized signaling as a critical component underlying this higher order process.

### Spatiotemporal differences

Many investigators have used DHPG to activate “chemical” LTD especially in hippocampal slices (25). More specifically, we have shown that DHPG activates only cell surface mGlu_5_ (16, 26, 27) by comparing the structure, membrane-permeability, transportability, binding curves, functional uptake, and Ca^2+^ release properties of this compound (15). In contrast, synaptic release of glutamate or Quis treatment, both of which are transported into the cell, constitutes a more realistic, full-fledged mGlu_5_ response. One surprise is that many of the steps outlined previously in the hippocampus using DHPG to induce mGlu_5_-LTD are performed by intracellular mGlu_5_ receptors in the striatum. Besides spatial differences, temporal signaling patterns are also triggered by the restricted mGlu_5_ receptor pools. For example, DHPG activation of cell surface mGlu_5_ elicited a rapid, transient Ca^2+^ response, whereas Quis activation of intracellular mGlu_5_ in striatal, cortical, and spinal cord dorsal horn neurons produced sustained Ca^2+^ responses (26, 27, 48). Because sustained responses vary in their diminution, exact “fold differences” are hard to measure; however, the peak amplitude of mGlu_5_-induced nuclear Ca^2+^ was ∼40% higher and 9-fold greater than surface mGlu_5_ Ca^2+^ transients in spinal cord dorsal horn neurons (48). Similarly, the peak amplitude of intracellular mGlu_5_ in striatal medium spiny neurons was ∼50% higher than DHPG at the initial peak and >5-fold greater, measuring the area under the Ca^2+^ curve from initiation outwards to 5 min (n = 100 neurons). These examples re-inforce the notion that mGlu5 can signal from different membrane platforms using different effector molecules to generate downstream sequelae with unique spatiotemporal profiles.

### Synaptic plasticity

Functionally, sustained intracellular GPCR signaling is often associated with long-term physiological processes. For example, sustained signaling following activation of many intracellular GPCRs can lead to increased transcription, proliferation, and cell survival (49, 50, 51). Prolonged mGlu_5_ Ca^2+^ signaling led to enhanced transcription particularly of synaptic plasticity genes (40). Another consequence might be increased localized translation (52). For example, both long-term potentiation and LTD require proteome remodeling (53), which comes about by localizing and translating mRNAs in axons and dendrites (18, 54, 55, 56, 57, 58, 59, 60). Locally translated mRNAs include Arc (9, 11, 61, 62, 63), PSD-95 (42, 64), and FMRP (64, 65). As shown here, Arc, PSD-95, and FMRP all play a role in striatal mGlu_5_-LTD following activation of intracellular mGlu_5_. Intriguingly, Schuman and colleagues (66) found that induction of mGlu_1/5_-LTD resulted in decreased mRNA motility leading to its enrichment near dendritic spines and enhanced mRNA translation. Although we have not explored mechanisms underlying mRNA motility in our system, potentially the intracellular mGlu_5_ receptor pool controls this process since only its activation leads to changes in Arc, FMRP, and PDS-95. mGlu_5_-LTD also requires actin remodeling (64, 67). Although a single pool of receptors might perform both functions, it is also possible that cell surface mGlu_5_ involves modulation of the actin cytoskeleton, whereas the intracellular pool contributes to mRNA immobility and increased protein synthesis. This conjecture is supported by earlier data in which DHPG-LTD was blocked in hippocampal slices by treatment with an actin-stabilizing drug (21, 68). The same agent blocked DHPG-mediated AMPA receptor internalization (21, 68).

**Figure 8:**
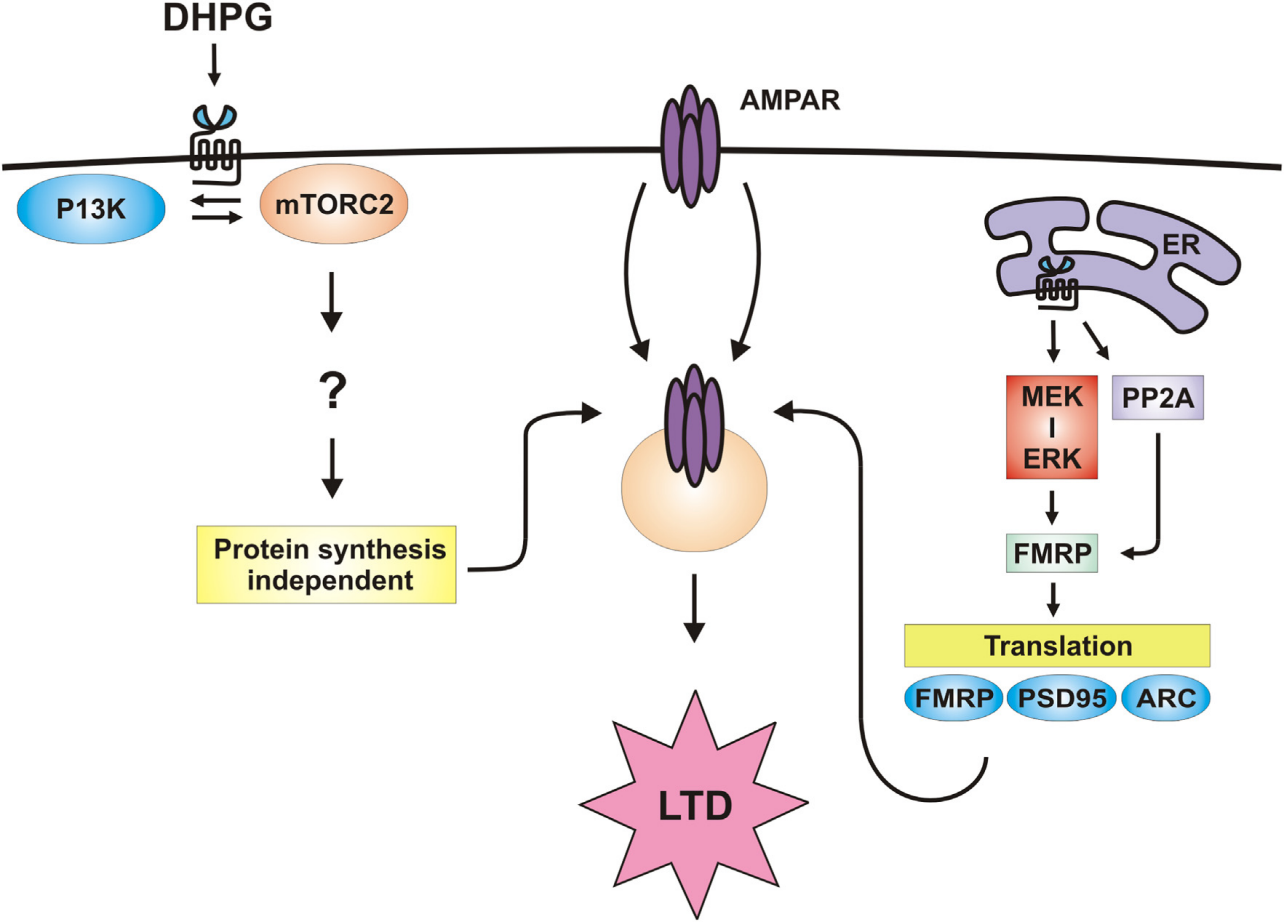
**Proposed model: Intracellular and cell surface receptors induce LTD by activating AMPAR internalization *via* divergent signaling pathways in striatum.** Effectors downstream of mammalian target of rapamycin complex 2 (mTORC2) mediating DHPG-LTD are as yet unknown. Phosphatidylinositol-3-kinase (PI3K), mTORC2, α-amino-3-hydroxy-5-methyl-4-isoxazolepropionic acid receptor (AMPAR), endoplasmic reticulum (ER), mitogen-activated protein kinase kinase (MEK), extracellular signal-regulated kinase (ERK), protein phosphatase 2 (PP2A), fragile X mental retardation protein (FMRP), post-synaptic density protein 95 (PSD-95), activity-regulated cytoskeleton-associated protein (Arc). LTD, long-term depression.

Although previous reports of hippocampal DHPG-LTD suggested an mTORC1-, protein-synthesis–dependent process (28, 69), recent studies using conditionally deleted accessory proteins such as Raptor (associated with mTORC1) and Rictor (associated with mTORC2) demonstrated that only Rictor deletion impaired DHPG-LTD (43). Our findings that striatal DHPG-LTD was mTORC2-dependent is in keeping with the Zhu *et al*. (43) report for hippocampal slices but anomalous given the lack of dependence upon new protein synthesis (Fig. 5). Intriguingly, protein-synthesis–independent LTD has been reported including in models of DHPG-LTD (70, 71, 72). In particular, the mitogen-activated protein kinase, p38, and its target MAPK-activated protein kinase 2 (MK2) are thought to regulate this form of synaptic plasticity in the hippocampus by depolymerizing the actin cytoskeleton resulting in dendritic spine loss and AMPA receptor internalization (71, 73). Other processes that might contribute to protein synthesis–independent mGlu_5_-LTD include the recently described mechanism by which BRAG2, the GDP/GTP exchange factor for ARF6, a small GTPase involved in membrane trafficking, interacts with PSD-95 and endophilin 3 to internalize AMPA receptors *via* clathrin-coated pits (74). Yet a third possibility builds on a recent report using 2-photon fluorescence lifetime imaging microscopy and optical reporters of PKA activity to show that DHPG activation of mGlu_5_ increases PKA activity (75, 76). The latter leads to GluA2 Ser880 phosphorylation and subsequent AMPA receptor internalization. Typically, PKA activation is due to Gs modulation; however, Chen *et al*. (76) demonstrated that Gq signaling is both sufficient and necessary to increase PKA activity. This is in keeping with our finding that the Gq inhibitor FR900359 (77) inhibits both DHPG- and Quis-mediated signaling in our experiments (not shown). A framework for current and future studies of striatal mGlu5-LTD is shown in Figure 8.

### Striatal plasticity

The striatal network receives inputs from many sources including sensory-motor cortex, various limbic structures, and associative areas (78). In addition, changes in striatal plasticity are influenced by dopamine release from SNc/VTA projections that modulate striatal medium spiny neurons (79). Dopamine can differentially modulate D1- and D2-expressing medium spiny neurons to release endogenous endocannabinoids (eCB), which act at presynaptic CB1 receptors inhibiting glutamate release. Interestingly, different stimulation protocols in the dorsal lateral striatum induce LTD by differentially mobilizing the eCBs, N-arachidonoylethanolamine, or 2-arachidonoylglycerol. In particular, using a novel genetically encoded fluorescent eCB biosensor, GRAB_eCB2.0_, to monitor eCB production, Liput *et al*. (80) demonstrated that activation of mGlu_1/5_, as well as other receptors, can trigger 2-arachidonoylglycerol mobilization through distinct mechanisms. These results suggest that different modes of eCB signaling can be generated depending on the amount of neural activity. This, in turn, might differentially activate cell surface or intracellular mGlu_5_ receptor pools, which might generate unique eCBs. Inasmuch as the striatum is highly involved in goal-directed and habitual motor control (81, 82), knowledge of how synaptic transmission and synaptic plasticity controls these processes is critical.

Studies over the last two decades have identified functional GPCRs on endosomes, ER, nuclei, mitochondria, lysosomes, the trans-golgi network, and the Golgi. Recently, we tabulated ∼120 GPCRs that exhibit compartmentalized signaling. Depending upon their intracellular location, compartmentalized GPCRS control functions such as transcription, proliferation, and survival, as well as metabolic, physiological processes, respiration, and apoptosis (49, 50, 51). While most of the details of these processes remain to be discovered, it makes sense that GPCRs, the master regulators of cellular function, maintain and regulate the complex spatial and temporal interactions occurring inside the cell as well as transmit signals from the outside. The current data further emphasize that mGlu_5_-localized signaling is a critical component underlying processes of synaptic plasticity underlying learning and memory. In-depth study of compartmentalized signaling and associated drug discovery studies will provide valuable insights and new location-specific drug targets.

## Experimental procedures

### Materials

(+)-α-amino-3,5-dioxo-1,2,4-oxadiazolidine-2-propanoic acid, Quis, DHPG, MPEP, CPCCOEt, (±)-4-(4-aminophenyl)-1,2-dihydro-1-methyl-2-propylcarbamoyl-6,7-methylenedioxyphthalazine (SYM2206), d-2-amino-5-phosphonopentanoic acid (APV), CNQX, 2-amino-(N,N)-1-bis(hexadecahydro-6,13-diisopropyl-2,5,9-trimethyl-1,4,7,11,14-pentaoxo-1H-pyrrolo[2,1]-[1,4,7,10,13] oxatetraazacyclohexadecin-10-yl)-4,6-dimethyl-3-oxo-3H-phenoxazine-1,9-dicarboxamide (actinomycin D), (2R,3S,4S)-2-[(4-methoxyphenyl)methyl]-3,4-pyrrolidinediol 3-acetate (anisomycin), 4-[2-(3,5-dimethyl-2-oxo-cyclohexyl)-2-hydroxyethyl]-2,6-piperidinedione (cycloheximide), N-[2-[[[3-(4-chlorophenyl)-2-propenyl]methylamino]methyl]phenyl]-N-(2-hydroxyethyl)-4-methoxybenzenesulphonamide (KN93), *N*-[(phenylmethoxy)carbonyl]-L-leucyl-N-[(1S)-1-formyl-3-methylbutyl]-L-leucinamide (MG132), 1,4-ciamino-2,3-dicyano-1,4-bis[2-aminophenylthio]butadiene (U0126), (1S,6bR,9aS,11R,11bR) 11-(acetyloxy)-1,6b,7,8,9a,10,11,11b-octahydro-1-(methoxymethyl)-9a,11b-dimethyl-3H-furo[4,3,2-de]indeno[4,5,-h]-2-h]-2-benzopyran-3,6,9-trione (wortmannin) and (3S,6R,7E,9R,10R,12R,14S,15E,17E,19E,21S,23S,26R,27R,34aS)-9,10,12,13,14,21,22,23,24,25,26,27,32,33,34,34a-hexadecahydro-9,27-dihydroxy-3-[(1R)-2-[(1S,3R,4R)-4-hydroxy-3-methoxycyclohexyl]-1-methylethyl]-10,21-dimethoxy-6,8,12,14,20,26-hexamethyl-23,27-epoxy-3H-pyrido[2,1-c][1,4]oxaazacyclohentriacontine-1,5,11,28,29(4H,6H,31H)-pentone (rapamycin), 2,3-Dihydro-*N,N*-dimethyl-2-oxo-3-[(4,5,6,7-tetrahydro-1*H*-indol-2-yl)methylene]-1*H*-indole-5-sulfonamide (SU6656) were purchased from Tocris (Bio-Techne Corporation). 2-amino-2-(3-cis/trans-carboxycyclobutyl)-3-(9H-thioxanthen-9-yl) propionic acid (LY393053) were obtained from Lilly Research Laboratories, Eli Lilly and Company.

### Cell cultures

Primary striatal cultures using neonatal 1-day old rat pups were prepared and maintained as previously described (16) The cells were plated onto 12-mm poly-d-lysine–coated glass coverslips (60,000/coverslip) for immunostaining or 12-well plates (10^6^ cells/well) for western blots. Cells were cultured in humidified air with 5% CO_2_ at 37 °C for 14 days before use. Generally, striatal neurons cultured for 14 days were preincubated with control medium containing mGlu_1_ antagonist CPCCOEt (20 μM) and AMPA receptor antagonist SYM2206 (25 μM) for 30 min at 37 °C before adding DHPG (100 μM) or Quis (20 μM) to measure mGlu_5_ specific activation. To evaluate the effects of different inhibitors on mGlu_5_ activation, the cells were also exposed to the inhibitor for 30 min before mGlu_5_ agonist application. These inhibitors include mGlu_5_ impermeable, nontransported antagonist, LY393053 (LY53, 20 μM), mGlu_5_ permeable antagonist, MPEP (10 μM), the translational inhibitor, cyclohexylamine (Cyc, 50 μM), the transcriptional inhibitor, actinomycin D (Act 10 μM), the PI3K inhibitor, wortmannin (Wor, 500 nM), CaMKII inhibitor, KN93 (10 μM), the MEK1/2 inhibitor, U0126 (1 μM), the PP2A inhibitor, okadaic acid (Oka, 5 nM), or the proteasome inhibitor, MG132 (10 μM).

### Immunocytochemistry

Primary striatal cultures at DIV 14 (days *in vitro*) after treatment were fixed and stained as described previously (26). Primary antibodies included rabbit monoclonal anti-mGlu_5_ (1:100; Abcam Inc), rabbit monoclonal anti-pAkt (1:100; Cell Signaling Technology Inc), rabbit polyclonal anti-Arc (1:400; Synaptic Systems), rabbit polyclonal anti-FMRP (1:250; Abcam Inc), mouse monoclonal anti-MAP2 (1:500; MilliporeSigma), rabbit monoclonal anti-PSD-95 (1:100; Cell Signaling Technology Inc), and mouse monoclonal anti-synapsin I (1:500; Thermo Fisher Scientific). Secondary antibodies included goat anti-mouse or anti-rabbit Cy3 (1:300; Jackson Immunoresearch) and goat anti-mouse or anti-rabbit Alexa 488 (1:300; Molecular Probes).

### Immunocytochemical detection of surface GluA2

Cells after treatment were fixed under nonpermeabilizing condition with 4% paraformaldehyde in PBS for 15 min immediately after treatment (83). Fixed cells were then washed with PBS and blocked with antibody dilution buffer (4% normal goat serum, 0.1% bovine serum albumin, 1× PBS, pH 7.4) buffer for 1 hour (84). For staining of surface AMPA receptors, cells were labeled with an antibody directed against the extracellular region of AMPA receptor subunit, GluA_2_ (mouse monoclonal 6C4, 1:100; Thermo Fisher Scientific) in antibody dilution buffer, washed, and saturated with Aelxa-488-conjugated secondary antibodies in antibody dilution buffer. The cells were then permeabilized with blocking buffer (1% bovine serum albumin, 0.25% milk powder/PBS) containing 0.3% Trition-X100 for 30 min and stained with rabbit monoclonal anti-mGlu_5_ (1:100; Abcam Inc). mGlu_5_ receptors were visualized by incubation with Cy3-conjugated secondary antibodies.

### Analysis of immunocytochemical data

Microscopy was performed with a Laser Confocal Microscope (Olympus BX 50WI) using an Olympus LUMPlanFl/lR 40×/0.80w or 60 ×/0.90w objectives. The images were collected by an Olympus Fluoview FVX Confocal Laser Scanning system using Fluoview 4.2 acquisition software (https://www.olympus-lifescience.com/en/downloads/detail-iframe/?0[downloads][id]=847249651). Images were processed with MetaMorph (version 7.7) (https://www.moleculardevices.com/products/cellular-imaging-systems/acquisition-and-analysis-software/metamorph-microscopy) Professional Image Analysis software, produced by Universal imaging. Immunofluorescence was analyzed around the cell bodies or along the proximal 40 μm of three or more dendrites per neuron. The automatic region function was used to generate polygons that surrounded soma and regions of the dendrite. The average intensity across all images in soma and neurites was calculated for each category treated with different agonists or antagonists and then compared. Separate controls were performed with each experiment, and a Student's *t* test was used to determine statistical significance.

### Biochemical measurements of surface-expressed GluA2

Biotinylation experiments were performed as previously described (15). Two-week-old cultured striatal neurons plated in 6-well plates (2 million/well, 3 wells per condition) in control medium containing AMPA/kainate receptor antagonist CNQX (20 μM), NMDA receptor antagonist D-AP5 (50 μM), and CPCCOEt (20 μM) were treated with DHPG (100 μM) or Quis (20 μM) for 15 min at 37 °C in the presence or absence of MEK1/2 inhibitor U0126 (10 μM), mTOR inhibitor rapamycin (1 μM), LY393053 (20 μΜ), or MPEP (10 μM). The cultures were then washed three times with ice-cold PBS (pH 8.0) and incubated with PBS (pH 8.0) containing 2 mM EZ-Link Sulfo-NHS-LC-Biotin (Thermo Fisher Scientific) at 4 °C for 30 min. Cultures were rinsed in PBS (pH 8.0) containing 100 mM glycine to quench the biotin reaction. Cultures were lysed in 100 μl/well modified RIPA buffer (50 mM Tris, pH 7.5, 150 mM NaCl, 1% NP-40, 0.1% SDS, 0.5% sodium deoxycholate, 1 mM PMSF, cOmplete protease inhibitor, and phosphatase inhibitor cocktail; MilliporeSigma). The homogenates were centrifuged at 14, 000*g* for 15 min at 4 °C. Twenty percent of the supernatant was removed to measure total GluA2. The remaining supernatant (80%) was incubated with NeutrAvidin agarose (Thermo Fisher Scientific) for 3 h at 4 °C, washed three times with RIPA buffer, and then bound proteins were resuspended in SDS sample buffer and heated at 55 °C for 15 min. Quantitative western blots were performed on both total and biotinylated (surface) proteins using anti-GluA_2_ (1:500, mouse monoclonal 6C4, Thermo Fisher Scientific).

### Western blotting

Western blotting was performed using whole cell extracts or biotinylated fractions from DIV 14 striatal cultures. Protein concentrations of whole cell lysates were determined using the Bradford assay (Bio-Rad). Proteins were separated by SDS-PAGE, blotted, and probed with mouse monoclonal anti-β-actin (1:2500; MilliporeSigma), rabbit monoclonal anti-Akt (1:500; Cell Signaling Technology, Inc), rabbit monoclonal anti-pAkt (1:1000; Cell Signaling Technology, Inc), Arc (1:1000; Synaptic Systems), rabbit polyclonal anti-FMRP(1:2000; Abcam Inc), rabbit polyclonal anti-pPP2A (Y307) (1;2000, Epitomics, Inc), rabbit monoclonal anti-RPS6 (1:1000; Cell Signaling Technology Inc), rabbit polyclonal anti-pRPS6 (1:500; Cell Signaling Technology Inc), and rabbit monoclonal anti-PSD-95 (1:500; Cell Signaling Technology Inc). A horseradish peroxidase conjugated with goat anti-rabbit IgG (1:2000; Cell Signaling Technology, Inc) or anti-mouse IgG (1:2000; MilliporeSigma) was used in conjunction with enhanced chemiluminescence (Bio-Rad) to detect the signal. Densitometric analyses were performed using the ChemiDoc MP system (Bio-Rad) together with associated Image Lab software (https://www.bio-rad.com/en-us/product/image-lab-software?ID=KRE6P5E8Z).

### PP2A enzyme activity profiling after DHPG or Quis treatment

Primary striatal neurons were pretreated with 20 μM CPCCOEt and 25 μM SYM2206 for 30 min, and followed by treatment for 0.5, 1, 2, 3, or 5 min with 100 μM DHPG or 20 μM Quis, an untreated sample was used as a control. Enzyme activity was measured as described by Mao *et al*. (31) with 4 × 10^5^ neurons/assay using a PP2A Immunoprecipitation Phosphatase Assay kit (MilliporeSigma). To immunoprecipitate PP2A, mouse antibodies against PP2Ac were added to a total of 150 μg/200 μl lysate, followed by 50% Protein A agarose/Sepharose bead slurry and incubation for 1 to 2 h at 4 °C. Beads were washed three times with PBS, followed by a single wash in assay buffer before the phosphopeptide was added to a final concentration of 0.75 mM and incubated for 10 min at 30 °C. Three independent experiments were performed, and the fold change at various time points was measured as the average absorbance value at: time = t (in minutes)/average absorbance, at t = 0. Student's *t* test was used to determine the significance of the fold change at different time points compared to t = 0.

### Protein synthesis

Dissociated striatal cultures at DIV 14 were radiolabeled with ^35^S-methionine and then treated with DHPG or Quis for 15 min. Cells were lysed in RIPA buffer with complete C protease inhibitor and EDTA. Protein lysates (30 μg/each well) were separated by SDS-PAGE. The gel was soaked in a solution containing 35% ethanol and 2% glycerol for 30 min and then dried with the application of heat (80 °C) and vacuum system. The gel was transferred onto the phosphorimager plate for 18 h and then developed on the Storm 860 Imager (Amersham Biosciences Corp).

### Electrophysiology

Mice (postnatal day 28–32) were anesthetized with isoflurane in a fume hood and decapitated. The frontal part of the hemisphere was pinned on a 3% agar base, and horizontal brain slices (400 μm thick) containing striatum and cortex were sectioned with a rotary slicer in artificial cerebrospinal fluid (ACSF) containing (in mM) the following: NaCl, 124; KCl, 5; CaCl_2_, 2; MgCl_2_, 2; NaHCO_3_, 22; NaH2PO_4_, 1.25; D-glucose, 10; fully gassed with a mixture of 95% O_2_/5% CO_2_ at 4 to 6 °C. Slices were placed in 10 ml beakers equipped with nylon mesh and allowed to equilibrate for at least 2 h in gassed ACSF at 30 °C. In some experiments, U0126 was added during this preincubation period. Individual slices were transferred to a recording chamber (2 ml) and perfused at a constant rate of 2 ml/min with gassed ACSF at 30 °C. PSs were induced by a bipolar stimulation electrode placed at the border of the dorsolateral striatum and the overlying white matter. PSs were monitored by applying single stimuli every 60 s at half-maximal intensity based on a control input-output (IO) curve. IO curves were determined using six different intensity stimuli before monitoring. After obtaining stable baseline recordings for at least 10 min, chemicals were administered. PSs were monitored by single stimuli once per minute or once per 5 minutes for at least 60 min after drug application. IO curves were repeated after 60 min for comparison to baseline. Statistical comparisons in electrophysiological studies were based on IO curves at baseline and 60 minutes after administration of DHPG or Quis to determine the degree of changes in the height of PS at the 50% maximal point. It should be noted that percentages are based on analysis of I/O curves rather than individual data points from each graph. Data were analyzed by one-way ANOVA followed by Dunnett’s multiple comparison test. For display purposes, graphs show data every 5 min.

### Animal studies

All animal procedures were performed according to NIH guidelines and approved by the Washington University Institutional Animal Care and Use Committee, protocols 21-0052 and 22-0228. Animals were under the care of the Washington University School of Medicine Division of Comparative Medicine.

## Data availability

Data used in this study are located in the article or are available upon request.

## Conflict of interest

C. F. Z. serves on the Scientific Advisory Board of Sage Therapeutics and has equity in the company. Sage Therapeutics was not involved in this work. The authors declare that they have no other conflict of interests with the contents of this article.
